# Linking the second messenger cyclic di-GMP signaling with the regulatory activity of the small RNA ErsA in *Pseudomonas aeruginosa*

**DOI:** 10.1093/nar/gkag930

**Published:** 2026-09-25

**Authors:** Silvia Ferrara, Tarcisio Brignoli, Silvia Santoro, Eloise Mastrangelo, Giovanni Bertoni

**Affiliations:** Department of Biosciences, Università degli Studi di Milano, Milan, 20133, Italy; Institute of Biophysics, National Research Council, Milan, 20133, Italy; Department of Biosciences, Università degli Studi di Milano, Milan, 20133, Italy; Department of Biosciences, Università degli Studi di Milano, Milan, 20133, Italy; Department of Biosciences, Università degli Studi di Milano, Milan, 20133, Italy; Institute of Biophysics, National Research Council, Milan, 20133, Italy; Department of Biosciences, Università degli Studi di Milano, Milan, 20133, Italy

## Abstract

Biofilm formation in *Pseudomonas aeruginosa* is regulated by intersecting signals from the envelope stress response and the cyclic di-GMP (c-di-GMP) second messenger network. We previously described that the envelope stress response sigma factor σ^22^ can simultaneously drive transcriptional activation and, via the small RNA ErsA, post-transcriptional repression of *algC*, which encodes a central enzyme in the production of sugar precursors for biofilm matrix exopolysaccharides. The inhibitory effect of ErsA on AlgC appeared incongruent with the well-established role of high c-di-GMP levels in promoting exopolysaccharide synthesis. In this study, we address this apparent contradiction by demonstrating that elevated c-di-GMP levels relieve ErsA-mediated repression of AlgC. This regulatory mechanism also applies to other ErsA targets, including the envelope homeostasis lipoprotein MlaA and the flagellar regulator FleN, which cooperates with FleQ to promote the biosynthesis of Pel/Psl exopolysaccharides and the CdrAB adhesins under high c-di-GMP conditions. Collectively, our results support a model in which ErsA serves as a central, c-di-GMP-responsive regulatory hub, linking envelope stress responses to c-di-GMP cascades to fine-tune key extracytoplasmic functions and coordinate biofilm formation.

## Graphical Abstract

**Graphical Abstract:**
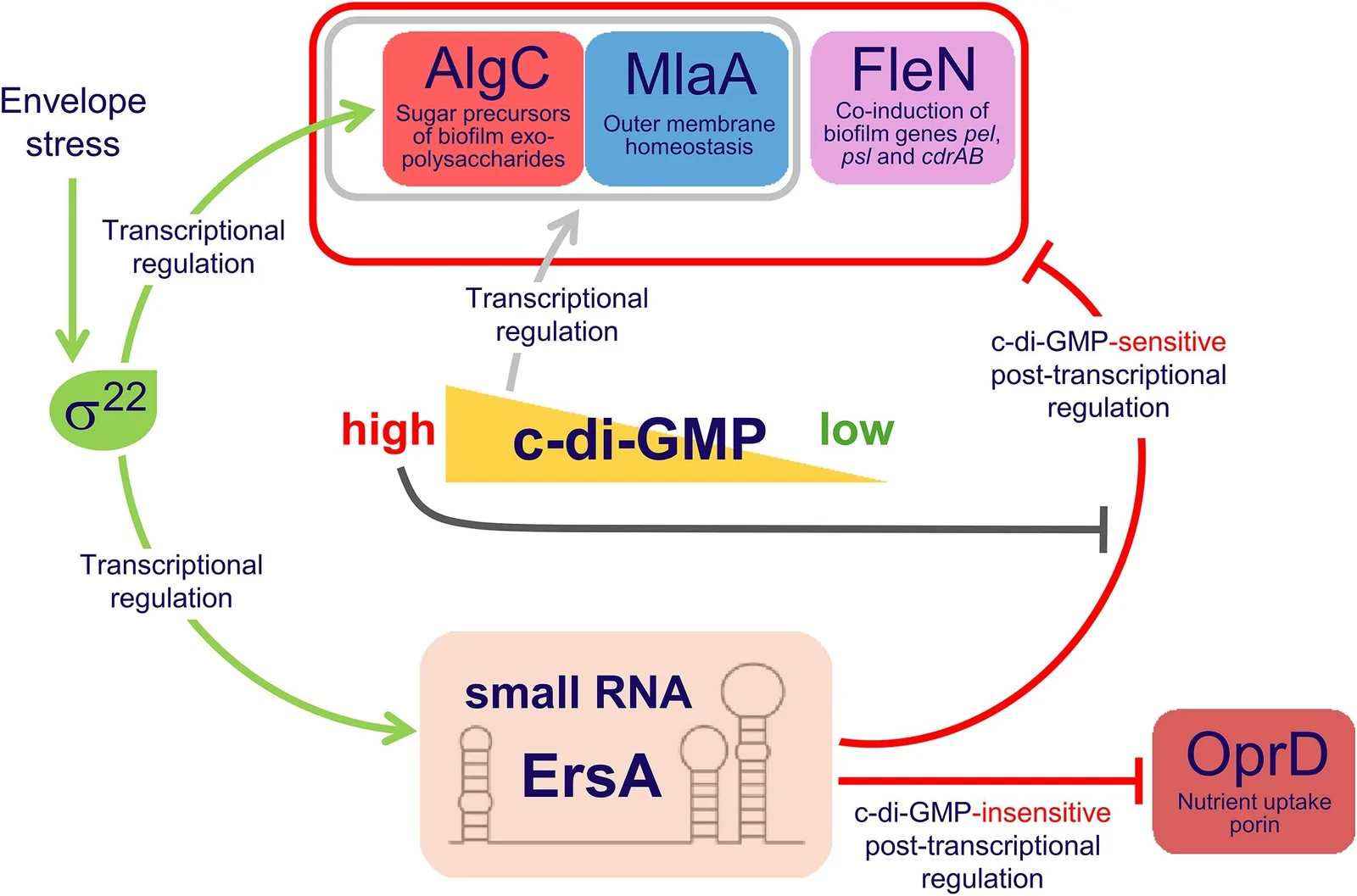

## Introduction

Regulatory programs involving the second messenger bis-(3′–5′) cyclic dimeric guanosine monophosphate (cyclic di-GMP or c-di-GMP) are widespread in bacteria and control diverse aspects of bacterial growth and behavior, including motility, pathogenesis, biofilm formation, and cell cycle progression [1–3]. All levels of regulation—transcriptional, post-transcriptional, and post-translational—can be targeted by c-di-GMP signaling pathways, which ultimately rely on effectors that bind to c-di-GMP. Several classes of c-di-GMP effectors have been identified [4], including proteins with diverse functions (e.g. transcription factors or enzymes) or RNA domains called riboswitches [5, 6]. A unique feature of c-di-GMP signaling is the presence, in most bacterial genomes, of numerous enzymes responsible for its synthesis and degradation [7]. C-di-GMP is synthesized by diguanylate cyclases (DGCs) with the catalytic activity residing in GGDEF domains and degraded by c-di-GMP-specific phosphodiesterases (PDEs) through the hydrolysis reaction by either EAL or HD-GYP domains. The balance between these opposing activities determines intracellular c-di-GMP levels, which in turn activate effector systems according to their binding affinity. Although the presence of multiple DGCs and PDEs suggests complex regulation, single-gene deletions often produce distinct phenotypes without altering global c-di-GMP pools [8]. This discrepancy challenges the model of exclusive global control [7–9]. Recent research demonstrates that bacteria flexibly integrate global regulation with highly localized c-di-GMP signaling [10].

In the opportunistic pathogen *Pseudomonas aeruginosa*, c-di-GMP is a major determinant of biofilm matrix production, coordinating the expression of multiple exopolysaccharide biosynthetic pathways, including Pel, Psl, and alginate, as well as extracellular DNA and matrix-stabilizing proteins such as adhesins CdrAB [11, 12]. The *pel, psl*, and *cdrAB* operons are transcriptionally induced under high c-di-GMP conditions [13–16]. Pel biosynthesis is also positively regulated by c-di-GMP at the post-translational level, with c-di-GMP binding to the synthase protein PelD and activating Pel polymerization [17, 18]. Similarly, alginate biosynthesis is positively regulated by c-di-GMP both post-translationally and transcriptionally. Upon binding of c-di-GMP to the Alg44 protein of the alginate synthase complex, alginate polymerization is activated [19, 20]. Furthermore, c-di-GMP positively regulates the transcription of the 12-gene alginate (*alg*) operon *algD*-*alg8*-*alg44*-*algKEGXLIJFA* [21]. Fluctuations in c-di-GMP levels exert opposite effects on the expression of flagellar genes and the *pel, psl*, and *cdrAB* operons. Under low c-di-GMP conditions, flagellar genes are upregulated while the *pel, psl*, and *cdrAB* operons are repressed. Elevated c-di-GMP levels reverse this expression profile. This molecular switch coordinates the transition from free-living planktonic cells to sessile biofilms and is driven mainly by the transcription factor FleQ and its associated regulator FleN [22].

The c-di-GMP signaling network in *P. aeruginosa* is remarkably complex, with over 40 genes predicted to encode DGCs or PDEs [23]. Functional screens of 41 mutants in the reference strain PAO1 showed that bacterial attachment and biofilm formation were the most impacted phenotypes, while motility was generally unaffected [24]. Interestingly, some mutants exhibited distinct phenotypic changes without significant variation in overall c-di-GMP levels, suggesting that both global and local c-di-GMP signaling contribute to biofilm regulation in *P. aeruginosa*.

In addition to c-di-GMP signaling, biofilm formation in *P. aeruginosa* is also influenced by the envelope stress response mediated by the alternative sigma factor σ^22^ (AlgT/U). The biosynthetic pathways for alginate, Psl, Pel, as well as lipopolysaccharide and rhamnolipids, compete for common sugar precursors supplied by AlgC [25], a checkpoint enzyme encoded by the *algC* gene located outside the *alg* operon. In this scenario, AlgC expression levels are therefore critical for coordinating the total amount and flux of the sugar precursor pool across multiple exopolysaccharide biosynthetic pathways. Albeit with different mechanisms [26, 27], both the *algC* gene and the *alg* operon are under the positive control of the sigma factor σ^22^. Superimposed on this control, we previously showed that *algC* is negatively regulated post-transcriptionally by the small RNA (sRNA) ErsA [28], which itself is positively regulated by σ^22^ [28]. Thus, σ^22^ can simultaneously converge transcriptional activation and post-transcriptional repression toward AlgC. We previously proposed that this regulatory architecture forms an incoherent feed-forward loop (I-FFL) enabling fine-tuned control of AlgC expression [28]. However, the ErsA-mediated repressive arm of this I-FFL acting on AlgC may become a limiting constraint under elevated c-di-GMP conditions that stimulate biofilm matrix production, as it could restrict the synthesis of precursor sugars. In addition, a σ^22^-mediated envelope stress response during biofilm matrix biogenesis would further enhance ErsA accumulation, thereby intensifying the constraint on the precursor pool.

Based on these considerations, we hypothesized that elevated intracellular c-di-GMP levels counteract the ErsA-mediated repression, thereby unlocking AlgC production when matrix synthesis is required. To test this hypothesis, we examined whether c-di-GMP affects ErsA at the transcriptional level and/or modulates its post-transcriptional regulatory activity. Here, we show that while c-di-GMP intracellular fluctuations do not alter ErsA steady-state levels, high c-di-GMP levels effectively deactivate ErsA-mediated post-transcriptional repression of AlgC. In parallel, we show that *algC* transcription is induced under high c-di-GMP conditions, resulting in coordinated expression with that of the *alg, pel*, and *psl* operons for the biogenesis of exopolysaccharide components of biofilm. Thus, c-di-GMP serves as a dual-level positive signal for AlgC expression, operating at both transcriptional and post-transcriptional tiers.

Furthermore, we identified and validated the *mlaA* gene—which encodes the outer membrane (OM) maintenance protein MlaA (also annotated as VacJ) [29, 30]—as a novel target of ErsA. Notably, *mlaA* displays a regulatory pattern similar to *algC*: it is transcriptionally induced by c-di-GMP, with ErsA-mediated repression restricted to low c-di-GMP levels. Finally, we identified an additional regulatory link between ErsA and c-di-GMP-dependent biofilm dynamics, showing that ErsA post-transcriptionally represses FleN. Collectively, these findings reveal a novel mode of regulatory crosstalk in which a global second messenger selectively relieves target-specific post-transcriptional constraint, and position ErsA as a central c-di-GMP-responsive hub coordinating σ^22^-mediated envelope stress responses with biofilm matrix biosynthesis and OM homeostasis.

## Materials and methods

### Bacterial strains and culture conditions

Bacterial strains used in this study are listed in Supplementary Table S1. *Pseudomonas aeruginosa* and *Escherichia coli* strains were routinely grown in Luria–Bertani broth (LB) at 37°C at 120 revolutions per minute (rpm) unless otherwise indicated. For selective *E. coli* growth, ampicillin, gentamicin, and chloramphenicol were added at 100, 20, and 25 μg/ml, respectively. For selective *Pseudomonas* growth, carbenicillin and gentamicin were added at 300 and 60 μg/ml, respectively, unless otherwise indicated. For the induction of the *P_BAD_* promoter of the vector plasmid pGM931, arabinose was added to a final concentration of 10 mM.

### Plasmids and mutant strains construction

Plasmids and oligonucleotides used in this study are listed in Supplementary Tables S1 and S2, respectively. Plasmids pBBR1-*mlaA::sfGFP*, pBBR1-*oprD::sfGFP*, and pBBR1-*fleN::sfGFP* expressing *mlaA::sfGFP, oprD::sfGFP*, and *fleN::sfGFP* translational fusions under the constitutive *P_LtetO-1_* promoter, respectively, were constructed as follows. A DNA fragment including the 79-nt UTR along with the first 35 codons (105 nt) of the *mlaA* open reading frame was polymerase chain reaction (PCR)-amplified from PAO1 genomic DNA with oligos 18/19, digested with *Nsi*I/*Nhe*I, and cloned into the sfGFP reporter vector pXG10-SF [31], giving rise to plasmid pXG10-*mlaA::sfGFP*. With the same procedure, a DNA fragment spanning the 78-nt leader of *oprD*, including the previously characterized ErsA binding site [32], and 90 nt of the *oprD* open reading frame (corresponding to 30 codons) was amplified with oligos 20/21, digested with *Nsi*I/*Nhe*I, and cloned into the sfGFP reporter vector pXG10-SF, giving rise to plasmid pXG10-*oprD::sfGFP*. Similarly, a DNA fragment encompassing the 112-nt leader region and the first 90 nt of the *fleN* coding sequence (30 codons) was amplified using oligos 22/23, digested with *Nsi*I/*Nhe*I, and cloned into pXG10-SF, generating pXG10-*fleN::sfGFP*. The DNA regions extending from the *P_LtetO-1_* promoter to the end of the sfGFP reporter gene were amplified by PCR from pXG10-*mlaA::sfGFP*, pXG10-*oprD::sfGFP*, and pXG10-*fleN::sfGFP* using oligos 24/25, digested with *Cla*I/*Xba*I, and cloned into the low-copy number shuttle vector pBBR1-MCS5 [33]. This gave rise to constructs pBBR1-*mlaA::sfGFP*, pBBR1-*oprD::sfGFP*, and pBBR1-*fleN::sfGFP*, respectively. To generate the plasmid pBBR1-*gfp*^C^, the *gfp*^C^ gene was amplified from pCdrA::*gfp*^C^ with oligos 16/17, digested with *Nhe*I/*Xba*I, and cloned into pXG10-SF, giving rise to pXG10-*gfp*^C^. The DNA fragment spanning from the *P_LtetO-1_* promoter to the end of the *gfp*^C^ reporter gene was amplified by PCR from pXG10-*gfp*^C^ with oligos 24/25 and cloned into pBBR1-MCS5. All constructs were verified by Sanger sequencing (Eurofins Genomics) using oligos 25 or 26. The plasmid pGM-*ersA*mut was generated with the same procedure used for pGM-*ersA* [28], using oligos 14/15 to amplify the mutant *ersA* allele.

*Pseudomonas aeruginosa* mutants in the *ersA* and *hfq* genes were generated using an enhanced markerless gene replacement method [34] as previously described for the construction of the Δ*ersA* [28] and Δ*hfq* [35] strains. PAO1 Δ*pp* Δ*ersA*, PAO1 Δ*pp* Δ*ersA yfiN*^ind^, and PAO1 Δ*pp* Δ*ersA* PA2133^ind^ mutants were generated from strains PAO1 Δ*pp*, PAO1 Δ*pp yfiN*^ind^, and PAO1 Δ*pp* PA2133^ind^, respectively. Allelic exchange resulted in an in-frame deletion spanning positions −46 to +82 relative to the *ersA* transcription start site [28]. The PAO1 Δ*pp* Δ*hfq* PA2133^ind^ mutant was generated from the PAO1 Δ*pp* PA2133^ind^ strain, resulting in an in-frame deletion of 65 codons within the *hfq* gene [35]. All plasmid constructs and deletion mutants were checked by sequencing with oligos indicated in Supplementary Table S2.

### RNA isolation and analysis

Total RNA was isolated from *P. aeruginosa* cultures as described previously [36]. Bacterial cells from 2–10 ml cultures were harvested by centrifugation, resuspended in RNAprotect Cell Reagent (Qiagen), incubated for 5 min at room temperature, pelleted by centrifugation, and stored at −80°C until use. Cell pellets were resuspended in TE–lysozyme buffer [10 mM Tris–HCl, 1 mM ethylenediaminetetraacetic acid (EDTA), 1 mg/ml lysozyme, pH 7.5], incubated at room temperature for 5 min, and lysed with QIAzol Lysis Reagent (Qiagen). Total RNA was extracted using the RNeasy Mini Kit (Qiagen) according to the manufacturer’s instructions, including RNase-free DNase I in-column treatment. Samples used for ErsA quantification were extracted with the miRNeasy kit (Qiagen) to enrich sRNAs (<200 nt), as described in the manufacturer’s protocol.

RNA concentration and purity were assessed using a BioPhotometer (Eppendorf). Only samples with an A_260_/A_280_ ratio ranging from 2.00 to 2.10 were used. RNA integrity was evaluated by electrophoresis on denaturing 7 M urea 6% acrylamide gels, confirming the absence of RNA degradation. To further minimize genomic DNA contamination, 5 μg of RNA were treated with RQ1 DNase (Promega) following the manufacturer’s instructions. Northern blot analyses were performed as described previously [36]. DNA oligonucleotide probes were 5′-end-labeled with [γ-^32^P]ATP (PerkinElmer, NEG502A) and T4 polynucleotide kinase (Promega, M4103) according to the manufacturer’s instructions. Oligos 1 and 3 were used to probe ErsA and 5S RNA, respectively. Radioactive bands were acquired after exposure to phosphor screens using a Typhoon™ 8600 Variable Mode Imager scanner (GE Healthcare BioSciences), visualized and quantified in triplicate with ImageQuant software (Molecular Dynamics). ErsA levels were calculated by normalization of ErsA bands to those of 5S RNA in the same lane. The resulting values were expressed as arbitrary units (AU).

Quantitative reverse transcription PCR (RT-qPCR) was performed as described previously [37]. Complementary DNA (cDNA) synthesis was carried out using 3 μg of DNase-treated RNA, SuperScript III Reverse Transcriptase (Invitrogen), and random hexamer primers (Invitrogen) according to the manufacturer’s instructions. No-reverse transcription (no-RT) controls were included for each sample to assess residual genomic DNA contamination. Successful reverse transcription was verified by PCR, using target-specific primers, confirming on agarose gels the presence of the expected band in cDNA samples and the absence of amplification in no-RT controls. RT-qPCR was performed using TB Green PCR Master Mix (Takara) in 15 μl reactions on a CFX Connect Real-Time System (Bio-Rad). Each reaction contained the cDNA template, gene-specific primers, and master mix according to the manufacturer’s recommendations. The oligo pairs used for amplification were as follows: 33/34 for *ersA*, 35/36 for *algC*, 37/38 for *mlaA*, 39/40 for *oprD*, and 41/42 for 16S. Primer specificity was verified by melting curve analysis, and no amplification was observed in negative controls. Thermal cycling conditions consisted of an initial denaturation at 95°C for 5 min followed by 39 cycles of amplification at 95°C for 15 s, 58°C for 20 s, and 72.5°C for 30 s. All reactions were performed in technical triplicate using RNA extracted from three independent biological replicates. The calculation of the relative expression of the analyzed genes in each strain was performed using the comparative *C*_t_ method [38]. Gene expression levels were normalized to 16S ribosomal RNA as the reference gene (Δ*C*_t_). For the overall comparison of messenger RNA (mRNA) levels, relative expression values were calculated as 2^−ΔCt^. Primer sequences and amplicon sizes are reported in Supplementary Table S2.

### Reporter-based evaluation of c-di-GMP levels

The evaluation of intracellular c-di-GMP levels was performed using wild-type strains PAO1 and PA14 and their corresponding *hfq* and *ersA* deletion mutants harboring the plasmid pCdrA::*gfp*^C^ [39]. The assays were performed either on liquid cell cultures or spotted cells grown on solid agar plates as described previously [40]. To test c-di-GMP levels on aerobically grown liquid cultures, strains were inoculated in the presence of gentamicin in 50-ml flasks filled with 10 ml of LB at an OD_600_ of 0.1 and grown at 37°C in a rotary shaker. Bacterial cells were collected at an OD_600_ of 2.0 by centrifugation, washed twice, and resuspended in phosphate buffered saline (PBS; 10 mM Na_3_PO_4_, 150 mM NaCl) to an OD_600_ of 1. Samples were then serially diluted 1.33-fold (corresponding to OD_600_ of 0.75, 0.5, and 0.25). Aliquots of 200 μl were transferred to black polystyrene 96-well microplates with a clear, flat bottom (Corning). At least three biological replicates were used for every experimental set. The absorbance (Abs_595_) and fluorescence intensity (FI_485/535_) were measured in an EnSight Multimode Plate Reader (PerkinElmer) using Kaleido data acquisition software. GFP activity was expressed in AU as FI_485/535_/Abs_595_. This assay on strains grown on solid substrate was performed by spotting 2 µl of bacterial cells at an OD_600_ of 0.1 on LB–agar plates with gentamicin. After overnight incubation at 37°C, the emitted fluorescence at 535 nm was captured after excitation at 485 nm with a ChemiDocTouch Imaging System (Bio-Rad) using ImageLab analysis software to generate qualitative images for fluorescence emission. Quantitative analysis was performed as described for bacteria grown in liquid culture, by collecting cells from solid agar plates, resuspending them in PBS, and following the same protocol.

### *In vivo* assays of sRNA/mRNA interactions

*In vivo* assays of sRNA/mRNA interactions were carried out as described previously [28, 41]. Briefly, strains carrying the *sfGFP* translational fusions with target genes or with *sfGFP* alone were inoculated in 15-ml tubes filled with 5 ml of LB at an OD_600_ of 0.1 and grown at 37°C in a rotatory shaker. Samples were taken after 6 and 24 h. The collected samples were centrifuged, washed twice, and resuspended in PBS (10 mM Na_3_PO_4_, 150 mM NaCl) to an OD_600_ of 1. Samples were then serially diluted 1.33-fold (corresponding to an OD_600_ of 0.75, 0.5, and 0.25). Aliquots of 200 μl were transferred to black polystyrene 96-well microplates with a clear, flat bottom (Corning). At least three biological replicates were used for every experimental set. The absorbance (Abs_595_) and fluorescence intensity (FI_485/535_) were measured in an EnSight Multimode Plate Reader (PerkinElmer) using Kaleido data acquisition software. GFP activity was expressed in AU as FI_485/535_/Abs_595_. Fluorescence intensity normalization to the corresponding pBBR1-*sf*GFP control—expressing *sf*GFP under the same promoter as the translational fusion constructs but lacking the target gene fragment—was performed to enable rigorous comparison between parental and Δ*ersA* c-di-GMP-dysregulated strains and to correct for baseline fluorescence variability. The results in these cases were expressed as a fluorescence ratio. No fluorescence intensity normalization to the corresponding pBBR1-*sf*GFP control was applied for ErsA-overexpressing strains, since ErsA overexpression had no detectable effect on *sf*GFP expression in any of the c-di-GMP backgrounds examined.

### *In vitro* assays of sRNA/mRNA interactions

RNAs used for *in vitro* interaction studies were generated by T7 RNA polymerase transcription from gel-purified DNA templates. DNA fragments for the preparation of ErsA, *algC, mlaA*, and *fleN* RNAs were amplified from *P. aeruginosa* PAO1 genomic DNA using oligo pairs 4/5, 6/7, 8/9 and 10/11, respectively. The DNA fragment for RseX RNA was amplified from *E. coli* C1a genomic DNA with oligos 12 and 13. *In vitro* transcription reactions were performed using the Riboprobe^®^ System-T7 (Promega) with 300 ng of DNA template. For radiolabeled probes, 50 μCi (α-^32^P) cytidine triphosphate (PerkinElmer) was included during transcription of *mlaA* and *fleN* RNAs. Synthesized RNAs were purified using the RNeasy MinElute Cleanup Kit (Qiagen), checked by denaturing polyacrylamide gel electrophoresis, and quantified using either an Eppendorf Biospectrometer or a Qubit fluorometer.

RNA–RNA interactions were analyzed by electrophoretic mobility shift assay (EMSA). Binding reactions were performed in a final volume of 10 μl containing 1× RNA-binding buffer (10 mM Tris–HCl, pH 7, 100 mM KCl, 10 mM MgCl_2_, 10% glycerol), and the indicated RNA species. For analysis of ErsA/*mlaA* and ErsA/*fleN* interactions, phospho-labelled *mlaA* or *fleN* mRNA (0.15 pmol) was incubated with increasing amounts of purified ErsA RNA (0, 0.08, 0.15, and 0.25 pmol) at 37°C for 20 min. Purified *E. coli* RseX RNA (0.25 pmol) was used as a negative control RNA. To investigate the effect of c-di-GMP on the ErsA/*algC* interaction, ErsA and *algC* RNAs were mixed at a fixed molar ratio of 2.5:1 (0.62 pmol ErsA and 0.25 pmol *algC*) in the presence of increasing amounts of c-di-GMP (0, 0.25, 25, 250, and 2500 μM, respectively). Following incubation at 37°C for 20 min, samples were resolved on native 6% polyacrylamide gels (acrylamide-bis ratio 29:1) prepared in 0.5× TBE buffer (45 mM Tris–borate, pH 8.0, 1 mM EDTA), using a Mini-Protean Electrophoresis System (Bio-Rad) at 4°C and 180 V for 90 min. For radiolabeled EMSAs, gels were dried and exposed to phosphor screens. Images were acquired using a Typhoon 8600 variable mode imager scanner (GE Healthcare BioSciences) and visualized with ImageQuant software (Molecular Dynamics). For chemiluminescent detection of ErsA-containing complexes, RNAs separated by native electrophoresis were transferred onto GeneScreen Plus nylon membranes (PerkinElmer) using a semi-dry electroblotter (Fastblot B33, Biometra) at 25 V and 400 mA for 1 h and subsequently ultraviolet (UV)-crosslinked using a Stratalinker 1800 UV Crosslinker (Stratagene). Membranes were hybridized with a biotinylated anti-ErsA probe (oligo 2) using the North2South Chemiluminescent Hybridization and Detection Kit (Thermo Scientific) according to the manufacturer’s instructions. After incubation with streptavidin–horseradish peroxidase conjugate, chemiluminescent signals were generated using luminol/enhancer and stable peroxide solutions and acquired with a ChemiDoc Touch Imaging System (Bio-Rad). Images were analyzed using ImageJ software.

### Liquid chromatography-tandem mass spectrometry-based evaluation of c-di-GMP levels

C-di-GMP extraction was performed using previously published protocols [42]. *Pseudomonas aeruginosa* cells in 5 ml of overnight culture were collected by centrifugation and resuspended in 300 µl of an ice-cold extraction solvent mixture (acetonitrile/methanol/water, 2/2/1, v/v/v). The suspension was incubated for 15 min at 4°C, then heated for 10 min at 95°C. After cooling on ice, the samples were centrifuged at max speed (14,680 rpm) for 5 min. The supernatant was set aside and kept on ice. The pellet was used for two further extractions, omitting the heating step, with 200 µl of extraction solvent. The three supernatants were combined (700 µl), and the solvent was evaporated in a SpeedVac vacuum concentrator (Thermo Fisher) at 40°C until dryness. The samples were resuspended in 100 µl of water by vortexing and subsequently centrifuged at 14,680 rpm for 5 min. The resulting supernatants were analyzed by high-resolution mass spectrometry using an Agilent 1290 Infinity II LC System (Agilent) coupled to a ZenoTOF 7600 System (SCIEX) equipped with Turbo V™ Ion Source with ESI Probe. Samples were separated on Luna Omega Polar C18 (1.6 µm, 2.1 × 100 mm) (Phenomenex). The temperature was set at 40°C. The analytes were eluted using a 5-min gradient from 100% buffer A (5 mM Ammonium Acetate in water) to 90% buffer B (acetonitrile) at a constant flow rate of 400 µl/min. MS spectra were collected over an m/z range of 250–750 Da in positive polarity, operating in MRM-HR mode (Multiple Reaction Monitoring – High Resolution) with collision energy set at 30. A 5-µl injection volume was analyzed in duplicate for each sample. Total protein concentration was determined for each sample from a 1-ml cell pellet harvested from the same culture. Cells were dissolved in 800 µl of 0.1 M NaOH at 95°C for 15 min, then protein concentration was determined using the Bicinchoninic Acid Protein Assay (G-Biosciences) following the manufacturer’s instructions. Relative quantification of the intracellular c-di-GMP content was obtained by normalizing the c-di-GMP peak area by the corresponding total protein concentration.

## Results

### ErsA steady-state levels are not influenced by c-di-GMP variations

To explore whether ErsA expression could be influenced by c-di-GMP states, we measured ErsA abundance in PAO1 Δ*pp yfiN*^ind^ and PAO1 Δ*pp* PA2133^ind^ strains. These are derivatives of PAO1 reference strain [43], which were engineered to maintain elevated or reduced c-di-GMP levels through arabinose–inducible expression of the DGC YfiN and the PDE PA2133, respectively [44, 45]. Both strains were derived from the parental PAO1 Δ*pp*, which lacks the first four genes of the *pel* and *psl* operons (Δ*pp*) to prevent cell aggregation and pellicle formation under high c-di-GMP levels [44, 45]. ErsA abundance was evaluated in these strains by RT-qPCR (Supplementary Fig. S1A) and Northern blot (Supplementary Fig. S1B and C). Notably, no significant differences in ErsA levels were observed in either the PAO1 Δ*pp yfiN*^ind^ or PAO1 Δ*pp* PA2133^ind^ strains compared to the parental PAO1 Δ*pp* control. These results show that ErsA expression is independent of c-di-GMP fluctuations.

### C-di-GMP modulates ErsA-mediated post-transcriptional regulation

Next, we investigated whether c-di-GMP levels influence the post-transcriptional regulatory activity of ErsA on *algC* mRNA. To test this, the PAO1 Δ*pp*, Δ*pp yfiN*^ind^, and Δ*pp* PA2133^ind^ strains and their respective Δ*ersA* derivatives were transformed with the reporter plasmid pBBR1-*algC::sfGFP*, which carries an *algC*-*sfGFP* translational fusion [28]. For ErsA overexpression tests, the Δ*pp*, Δ*pp yfiN*^ind^, and Δ*pp* PA2133^ind^ strains were co-transformed with pBBR1-*algC::sfGFP* and either the ErsA-expression vector pGM-*ersA* or the empty vector control pGM931 [28].

Before assessing *algC*-*sfGFP* translational fusion activity across the different c-di-GMP and genetic backgrounds, we performed preliminary control assays to validate the robustness of our experimental setup. First, we evaluated whether the lack of ErsA impacts c-di-GMP dysregulation in the above engineered strains. To achieve this, parental and Δ*ersA* strains were transformed with the reporter plasmid pCdrA::*gfp*^C^, which contains the c-di-GMP-inducible *cdrA* promoter transcriptionally fused to the green fluorescent protein *gfp^C^* gene [39]. As expected, reporter activity was elevated in the Δ*pp yfiN*^ind^ strain and reduced in the Δ*pp* PA2133^ind^ strain relative to the Δ*pp* baseline (Supplementary Fig. S2). Within both Δ*pp yfiN*^ind^ and Δ*pp* PA2133^ind^ backgrounds, reporter activity was strictly comparable between the parental and Δ*ersA* derivatives. This confirmed that ErsA absence does not interfere with engineered c-di-GMP dysregulation. Notably, we observed a 20% increase in reporter activity in the Δ*pp* Δ*ersA* mutant (Supplementary Fig. S2), preliminarily suggesting that ErsA exerts an effect on physiological c-di-GMP levels. Second, we confirmed that differences in c-di-GMP levels did not bias ErsA overexpression from the pGM-*ersA* plasmid; as shown in Supplementary Fig. S1B and C, ErsA overexpression levels remained comparable between the Δ*pp yfiN*^ind^ and Δ*pp* PA2133^ind^ strains. Third, to rule out nonspecific artifacts, we evaluated whether c-di-GMP levels, *ersA* deletion, or ErsA overexpression influenced *sf*GFP expression independently of the *algC* target sequence. To this end, our entire panel of strains—including the Δ*ersA* mutants and the ErsA-overexpressing variants—was transformed with the control plasmid pBBR1-*sf*GFP [28], which drives *sf*GFP expression from the same promoter as the *algC*-*sfGFP* fusion, but lacks the *algC* fragment. Control assays showed that while the genetic and c-di-GMP backgrounds caused fluctuations in baseline *sf*GFP fluorescence (Supplementary Fig. S3A), ErsA overexpression had no impact on *sf*GFP expression within any given c-di-GMP background (Supplementary Fig. S3B). Consequently, *algC::sf*GFP reporter activity with and without ErsA overexpression could be compared directly within each specific c-di-GMP context. Conversely, normalization to the corresponding pBBR1-*sf*GFP control was necessary for comparisons between parental and Δ*ersA* strains to account for baseline fluorescence variability (Supplementary Fig. S3A).

**Figure 1.**
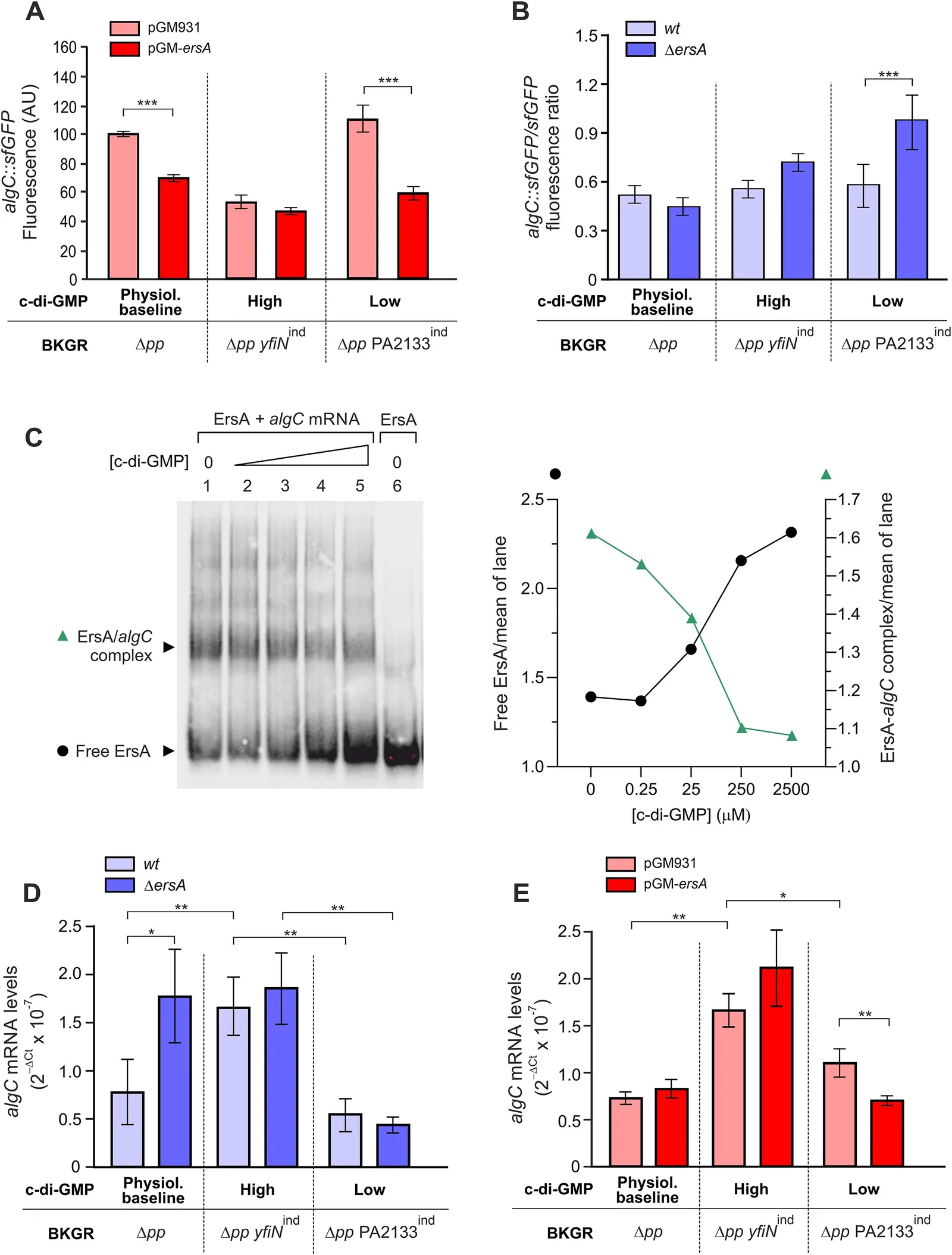
Influence of ErsA and c-di-GMP on the post-transcriptional regulation and steady-state mRNA levels of *algC*. The *in vivo* assays were conducted in the genetic backgrounds (BKGR) of PAO1 Δ*pp* and its derivatives PAO1 Δ*pp yfiN*^ind^ and PAO1 Δ*pp* PA2133^ind^ with dysregulated c-di-GMP levels. Specifically, the levels of c-di-GMP expressed by PAO1 Δ*pp yfiN*^ind^ and PAO1 Δ*pp* PA2133^ind^ are higher or lower, respectively, than those of their parental PAO1 Δ*pp* (physiological baseline). (**A**) Comparison of fluorescence resulting from the *P_LtetO-1_*-driven *algC::sfGFP* translational fusion of the plasmid pBBR1-*algC::sfGFP* combined with the overexpressing ErsA vector pGM-*ersA* or with the corresponding empty vector pGM931. (**B**) Comparison of fluorescence from pBBR1-*algC::sfGFP* normalized to that from pBBR1-*sfGFP* in the same background between wt and Δ*ersA* mutants. In panels (A) and (B), data are expressed as the mean (*n* = 12) ± standard deviation (SD). (**C**) *In vitro* interaction between ErsA RNA and *algC* mRNA by EMSA in the presence of increasing c-di-GMP concentrations (0, 0.25, 25, 250, 2500 μM: lanes 1, 2, 3, 4, 5, respectively; lane 6 is a control of ErsA RNA alone without c-di-GMP). Arrowheads indicate free ErsA RNA or ErsA/*algC* mRNA complexes. Signal quantification was performed with ImageJ, and band intensities were normalized to the mean intensity of the corresponding lane. (**D**) Comparison of *algC* mRNA levels determined by RT-qPCR and displayed graphically as 2^−ΔCt^ values in PAO1 Δ*pp*, PAO1 Δ*pp yfiN*^ind^, and PAO1 Δ*pp* PA2133^ind^ and their Δ*ersA* derivatives. (**E**) Comparison of *algC* mRNA levels determined by RT-qPCR and displayed graphically as 2^−Δ^*^C^*^t^ values in PAO1 Δ*pp*, PAO1 Δ*pp yfiN*^ind^, and PAO1 Δ*pp* PA2133^ind^ with the overexpressing ErsA vector pGM-*ersA* or with the corresponding empty vector pGM931, respectively. Statistical significance was calculated by one-way analysis of variance (ANOVA) with *post-hoc* Tukey’s honestly significant difference test (*P-value <.05; **P-value <.01; ***P-value <.001).

Following these setting controls, we could investigate whether c-di-GMP levels impact the post-transcriptional regulation of ErsA on *algC* mRNA. To assess this, we measured the reporter activity of the translational fusion *algC::sf*GFP across the different backgrounds. As shown in Fig. 1A, ErsA overexpression downregulated *algC* translation in the parental strain Δ*pp* and, to a greater extent, in the low c-di-GMP strain Δ*pp* PA2133^ind^. This effect was absent in the high c-di-GMP Δ*pp yfiN*^ind^ strain, suggesting that elevated c-di-GMP levels impair the ability of ErsA to repress *algC* mRNA. A similar trend emerged when ErsA was expressed at physiological levels. As shown in Fig. 1B, ErsA-mediated repression of *algC* appeared to be specifically effective at low c-di-GMP levels in Δ*pp* PA2133^ind^, as only under this condition did the deletion of ErsA result in a significant increase in reporter activity.

Based on these results, we hypothesized that c-di-GMP could interfere with the interaction between ErsA and *algC* mRNA. To test this, we performed EMSAs [28] to assess the interaction between ErsA RNA and *algC* mRNA in the presence of increasing amounts of c-di-GMP. As shown in Fig. 1C, c-di-GMP reduced ErsA/*algC* mRNA complex formation in a dose-dependent manner, with a reciprocal increase in free ErsA RNA. These *in vitro* results correlated with the impaired ErsA-mediated regulation of *algC* mRNA observed *in vivo* in the high c-di-GMP Δ*pp yfiN*^ind^ strain (Fig. 1A and B).

We next assessed whether the levels of c-di-GMP influence *algC* expression at the transcriptional level. To do this, we quantified *algC* mRNA abundance across the three c-di-GMP backgrounds, both wild-type and Δ*ersA*. As shown in Fig. 1D, Δ*pp yfiN*^ind^ expressed significantly higher *algC* mRNA levels than Δ*pp* PA2133^ind^, independent of the Δ*ersA* mutation. These results suggested that *algC* transcription is stimulated by c-di-GMP, consistent with the *alg* operon genes [21]. Notably, the Δ*ersA* mutation in the Δ*pp* background elevated *algC* transcript abundance (Fig. 1D), suggesting that ErsA regulates *algC* transcription under physiological c-di-GMP conditions. We also evaluated *algC* mRNA levels under ErsA overexpression (Fig. 1E). While ErsA overexpression did not alter *algC* mRNA levels in the high c-di-GMP Δ*pp yfiN*^ind^ strain, it significantly reduced transcript abundance in the low c-di-GMP Δ*pp* PA2133^ind^ background. This decrease is likely due to the well-known effect that a translationally repressed mRNA becomes more unstable, reinforcing the notion that the ErsA-mediated translational repression of *algC* mRNA is more pronounced at low rather than high c-di-GMP levels.

**Figure 2.**
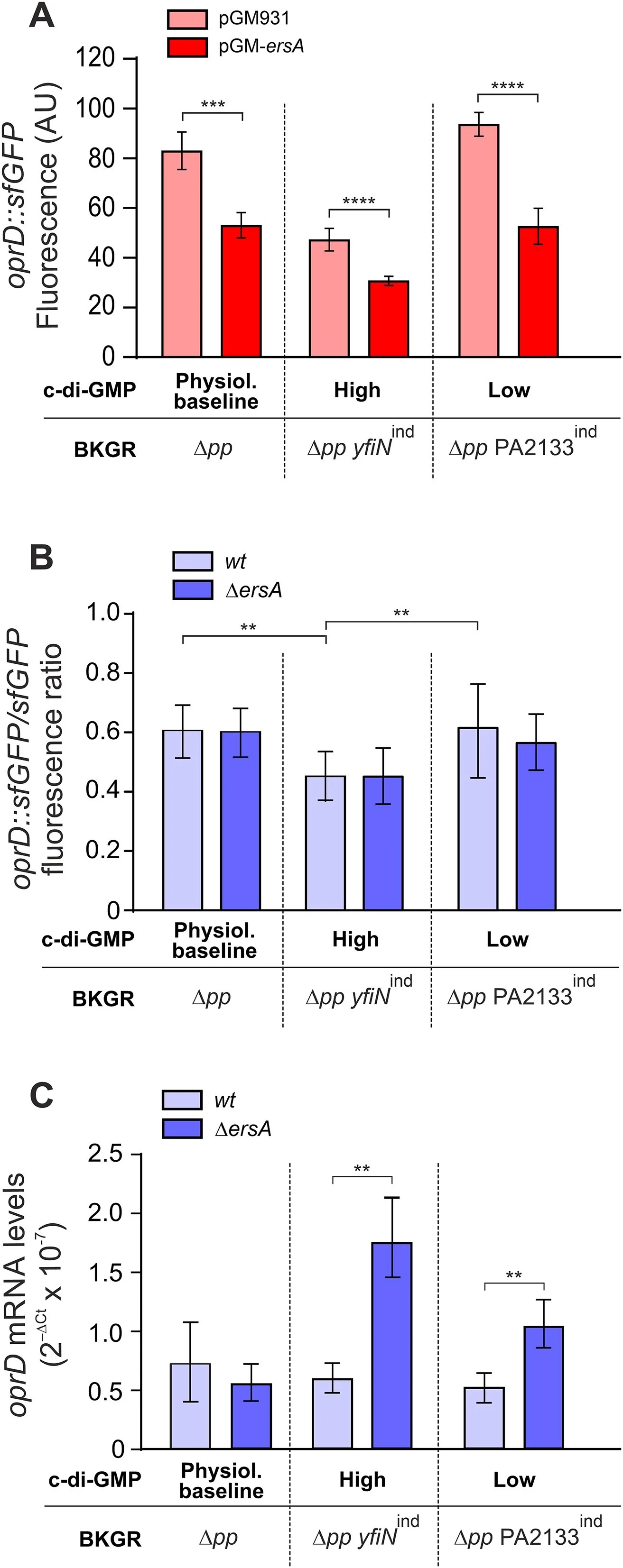
Influence of ErsA and c-di-GMP on the post-transcriptional regulation and steady-state mRNA levels of *oprD*. The *in vivo* assays were conducted in the genetic backgrounds (BKGR) of PAO1 Δ*pp* and its derivatives PAO1 Δ*pp yfiN*^ind^ and PAO1 Δ*pp* PA2133^ind^ with dysregulated c-di-GMP levels. Specifically, the levels of c-di-GMP expressed by PAO1 Δ*pp yfiN*^ind^ and PAO1 Δ*pp* PA2133^ind^ are higher or lower, respectively, than those of their parental PAO1 Δ*pp* (physiological baseline). (**A**) Comparison of fluorescence resulting from the *P_LtetO-1_*-driven *oprD::sfGFP* translational fusion of the plasmid pBBR1-*oprD::sfGFP* combined with the overexpressing ErsA vector pGM-*ersA* or with the corresponding empty vector pGM931. (**B**) Comparison of fluorescence from pBBR1-*oprD::sfGFP* normalized to that from pBBR1-*sfGFP* in the same background between wt and Δ*ersA* mutants. In panels (A) and (B), data are expressed as the mean (*n* = 12) ± SD. (**C**) Comparison of *oprD* mRNA levels determined by RT-qPCR and displayed graphically as 2^−Δ^*^C^*^t^ values in PAO1 Δ*pp*, PAO1 Δ*pp yfiN*^ind^, and PAO1 Δ*pp* PA2133^ind^ and their Δ*ersA* derivatives. Statistical significance was calculated by one-way ANOVA with *post-hoc* Tukey’s honestly significant difference test (*P-value <.05; **P-value <.01; ***P-value <.001; ^****^P-value <.0001).

To determine whether the effects of c-di-GMP on ErsA regulation were restricted to *algC* mRNA or extended to other ErsA targets, we tested another characterized target of ErsA, the *oprD* mRNA [32]. To this end, we constructed a set of strains as above with the reporter vector pBBR1-*oprD::sfGFP*, carrying an *oprD* translational fusion with the *sfGFP* gene. As shown in Fig. 2A, ErsA overexpression reduced *oprD* translation independently of the background c-di-GMP levels. This stands in contrast to *algC*, which exhibited no translational down-regulation under high c-di-GMP levels in the Δ*pp yfiN*^ind^ strain. Furthermore, different from *algC*, comparison of the parental and Δ*ersA* strains revealed no detectable ErsA-mediated regulation of *oprD* at physiological levels of ErsA, regardless of the c-di-GMP background (Fig. 2B). However, independently of ErsA, *oprD* translation is downregulated at high c-di-GMP levels, a result consistent with a previous report [46].

Analysis of transcript levels revealed a more complex regulatory pattern than that observed for *algC* (Fig. 2C). While c-d-GMP levels did not alter *oprD* mRNA abundance in the presence of ErsA, deletion of ErsA both in Δ*pp yfiN*^ind^ and Δ*pp* PA2133^ind^ resulted in elevated *oprD* mRNA levels. This suggests that, at opposing extremes of c-di-GMP levels, the lack of ErsA can lead to transcriptional deregulation of *oprD*.

### Testing ErsA regulation at low c-di-GMP levels reveals a novel target mRNA

**Figure 3.**
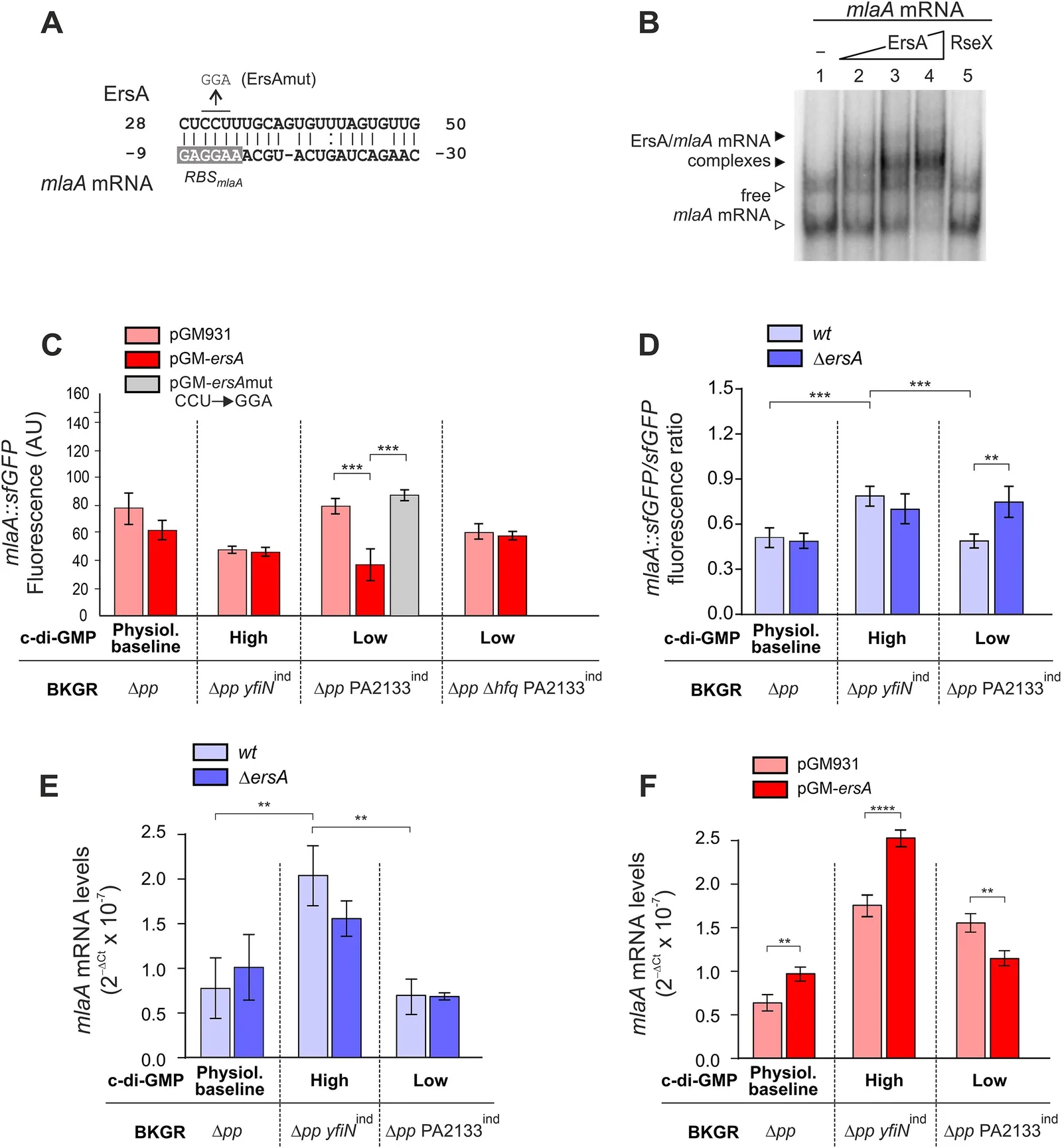
Influence of ErsA and c-di-GMP on the post-transcriptional regulation and steady-state mRNA levels of *mlaA*. (**A**) Base pairing between the leader sequence of *mlaA* mRNA (coordinates −9 to −30 from the AUG translation start site) and ErsA (from nucleotides 28 to 50) predicted by the web tool TargetRNA. The RBS of *mlaA* mRNA (*RBS_mlaA_*) and the base changes resulting in the ErsAmut variant are indicated. (**B**) *In vitro* analysis of the interaction between ErsA and *mlaA* mRNA by electrophoretic mobility shift. Increasing amounts of purified ErsA RNA (0, 0.08, 0.15, 0.25 pmol; lanes 1–4) or, as a negative control, *E. coli* RseX RNA (0.25 pmol; lane 5) were incubated at 37°C for 20 min with 0.15 pmol of radiolabeled *mlaA* mRNA and loaded onto a native 6% polyacrylamide gel. White and black arrowheads indicate free *mlaA* mRNA and ErsA/*mlaA* mRNA complexes, respectively. The *in vivo* study of the post-transcriptional regulation and steady-state mRNA abundance of *mlaA* at different levels of intracellular c-di-GMP was conducted in the same way as for *algC* and *oprD*, i.e. in the genetic backgrounds (BKGR) of PAO1 Δ*pp* (c-di-GMP physiological baseline) and its derivatives PAO1 Δ*pp yfiN*^ind^ (high c-di-GMP) and PAO1 Δ*pp* PA2133^ind^ (low c-di-GMP), in the presence of ErsA overexpression or the Δ*ersA* mutation. (**C**) Comparison of fluorescence resulting from the *P_LtetO-1_*-driven *mlaA::sfGFP* translational fusion of the plasmid pBBR1-*mlaA::sfGFP* combined with the overexpressing ErsA vectors pGM-*ersA*, pGM-*ersA*mut, or with the corresponding empty vector pGM931. The analysis was also performed in PAO1 Δ*pp* Δ*hfq* PA2133^ind^. (**D**) Comparison of fluorescence from pBBR1-*mlaA::sfGFP* normalized to that from pBBR1-*sfGFP* in the same background between wt and Δ*ersA* mutants. In panels (C) and (D), data are expressed as the mean (*n* = 12) ± SD. (**E**) Comparison of *mlaA* mRNA levels determined by RT-qPCR and displayed graphically as 2^−Δ^*^C^*^t^ values in PAO1 Δ*pp*, PAO1 Δ*pp yfiN*^ind^, and PAO1 Δ*pp* PA2133^ind^ and their Δ*ersA* derivatives. (**F**) Comparison of *mlaA* mRNA levels determined by RT-qPCR and displayed graphically as 2^−Δ^*^C^*^t^ values in PAO1 Δ*pp*, PAO1 Δ*pp yfiN*^ind^, and PAO1 Δ*pp* PA2133^ind^ with the overexpressing ErsA vector pGM-*ersA* or with the corresponding empty vector pGM931, respectively. Statistical significance was calculated by one-way ANOVA with *post-hoc* Tukey’s honestly significant difference test (**P-value <.01; ***P-value <.001; ^****^P-value <.0001).

The observation that ErsA-mediated translational regulation of *algC* is modulated by c-di-GMP levels led us to reconsider *mlaA*, a gene encoding an integral protein of the OM, as a potential target of ErsA. The *mlaA* mRNA was previously predicted as a target of ErsA in our *in silico* analyses with the web tool TargetRNA [47], which also correctly predicted *algC* as a direct ErsA target [28]. TargetRNA suggested an interaction between ErsA, from nt 28 to 50 in the region located between the stem and loops SL1 and SL2, and the 5′ UTR of the *mlaA* mRNA spanning from nucleotide −9 to −30 from the AUG translation start codon (Fig. 3A). This interaction was validated *in vitro* with purified components (Fig. 3B). Since base-pairing with ErsA involves the ribosome binding site (RBS) of *mlaA* mRNA, we hypothesize a negative regulatory role of ErsA on *mlaA*. However, assays with a *mlaA::sfGFP* translational fusion using the plasmid *pBBR1-mlaA::sfGFP* in PAO1, with ErsA overexpression or comparing PAO1 with PAO1 Δ*ersA*, failed to validate any ErsA-mediated regulation of *mlaA* (data not shown). The evidence that ErsA-mediated regulation can depend on c-di-GMP levels prompted us to repeat the assays with pBBR1-*mlaA::sfGFP* in the different c-di-GMP backgrounds used above. As shown in Fig. 3C, ErsA overexpression downregulated *mlaA* in Δ*pp* PA2133^ind^ but not in Δ*pp* and Δ*pp yfiN*^ind^. Consistent with the prediction of the interaction between ErsA and *mlaA* mRNA shown in Fig. 3A, the downregulation was abolished when the CCU → GGA mutation, which impairs ErsA base pairing with the RBS of *mlaA*, was introduced into ErsA (Fig. 3C). Furthermore, overexpression of ErsA in the *hfq* mutant of Δ*pp* PA2133^ind^ had no effect (Fig. 3C), suggesting that the ErsA-mediated downregulation of *mlaA* mRNA is Hfq-dependent. As observed for *algC*, physiological levels of ErsA were sufficient to downregulate *mlaA* mRNA specifically at low levels of c-di-GMP in Δ*pp* PA2133^ind^, as evidenced by the significant increase in reporter activity seen only in the corresponding Δ*ersA* mutant (Fig. 3D). These results indicate that, similar to *algC*, the ErsA-mediated repression of *mlaA* mRNA translation is effective under low c-di-GMP levels. However, *mlaA* exhibited two notable differences compared to *algC*: (i) the c-di-GMP levels in Δ*pp* were high enough to inhibit the repression of *mlaA* by ErsA and (ii) *mlaA* appeared more translated at high c-di-GMP levels in Δ*pp yfiN*^ind^ strain (Fig. 3D).

As with *algC* and *oprD*, we investigated whether c-di-GMP levels influence *mlaA* at the transcriptional level. As shown in Fig. 3E, Δ*pp yfiN*^ind^ expressed much higher levels of *mlaA* mRNA than Δ*pp* PA2133^ind^ regardless of the Δ*ersA* mutation. Different from *algC*, the lack of ErsA in the Δ*pp* background did not elevate *mlaA* transcript abundance (Fig. 3E). The overexpression of ErsA reduced the *mlaA* mRNA amounts in Δ*pp* PA2133^ind^ (Fig. 3F), consistent with the results for *algC* (Fig. 1E). Unexpectedly, in Δ*pp* and Δ*pp yfiN*^ind^ strains, overexpression of ErsA increased *mlaA* mRNA amounts (Fig. 3F). This suggests that induction of high ErsA levels in the presence of increased c-di-GMP may stimulate *mlaA* mRNA stability and/or transcription.

### FleN is an additional target of ErsA post-transcriptional regulation

Our results indicated that c-di-GMP levels can influence ErsA-mediated regulatory activity. However, they also pointed to a potential feedback loop wherein ErsA influences c-di-GMP levels. This was evidenced by a 20% higher activity of the *cdrA* promoter in the plasmid pCdrA::*gfp*^C^ upon deleting *ersA* in the PAO1 Δ*pp* background (Supplementary Fig. S2). To further investigate the impact of ErsA on c-di-GMP levels, PAO1 and PAO1 Δ*ersA* were transformed with the plasmid pCdrA::*gfp*^C^. To preliminarily verify that the translation of the *gfp*^C^ reporter gene was not affected by the *ersA* deletion, we generated the plasmid pBBR1-*gfp*^C^ in which the *gfp*^C^ gene is transcriptionally fused to the constitutive *P_LtetO-1_* promoter. Fluorescence from this plasmid was comparable in the two genetic backgrounds, wild-type and Δ*ersA* (Supplementary Fig. S4).

**Figure 4.**
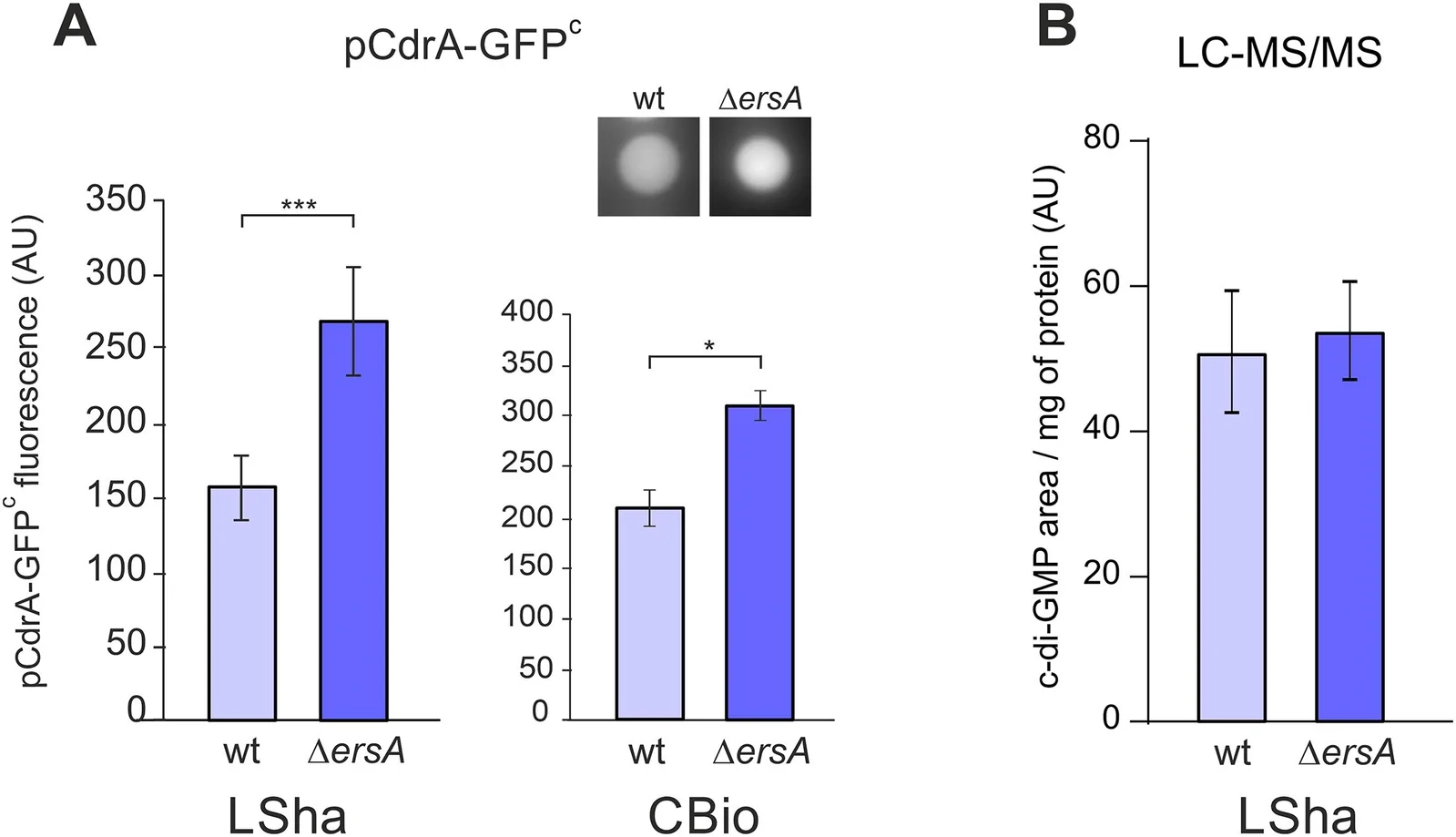
ErsA influence on *cdrA* promoter and on intracellular c-di-GMP levels in *P. aeruginosa* PAO1. (**A**) Quantitative evaluation of fluorescence levels produced by PAO1 or PAO1 Δ*ersA* cells with the reporter plasmid pCdrA::*gfp*^C^ grown in liquid LB medium with shaking (LSha) or on solid LB agar plates (CBio). Values are expressed in AU and represent the mean ± SD of three biological replicates. Upper images of the fluorescence emitted by cell spots are representative of one of the three biological replicates. (**B**) Assessment of intracellular c-di-GMP levels by LC-MS/MS in PAO1 or PAO1 Δ*ersA* cells grown in liquid LB medium with shaking (LSha). Values are expressed as the ratio between the peak area corresponding to c-di-GMP and the protein content of the sample and represent the mean ± standard error of the mean of four biological replicates. Statistical analysis has been performed using Welch’s *t*-test (*P-value <.05; ***P-value <.001).

**Figure 5.**
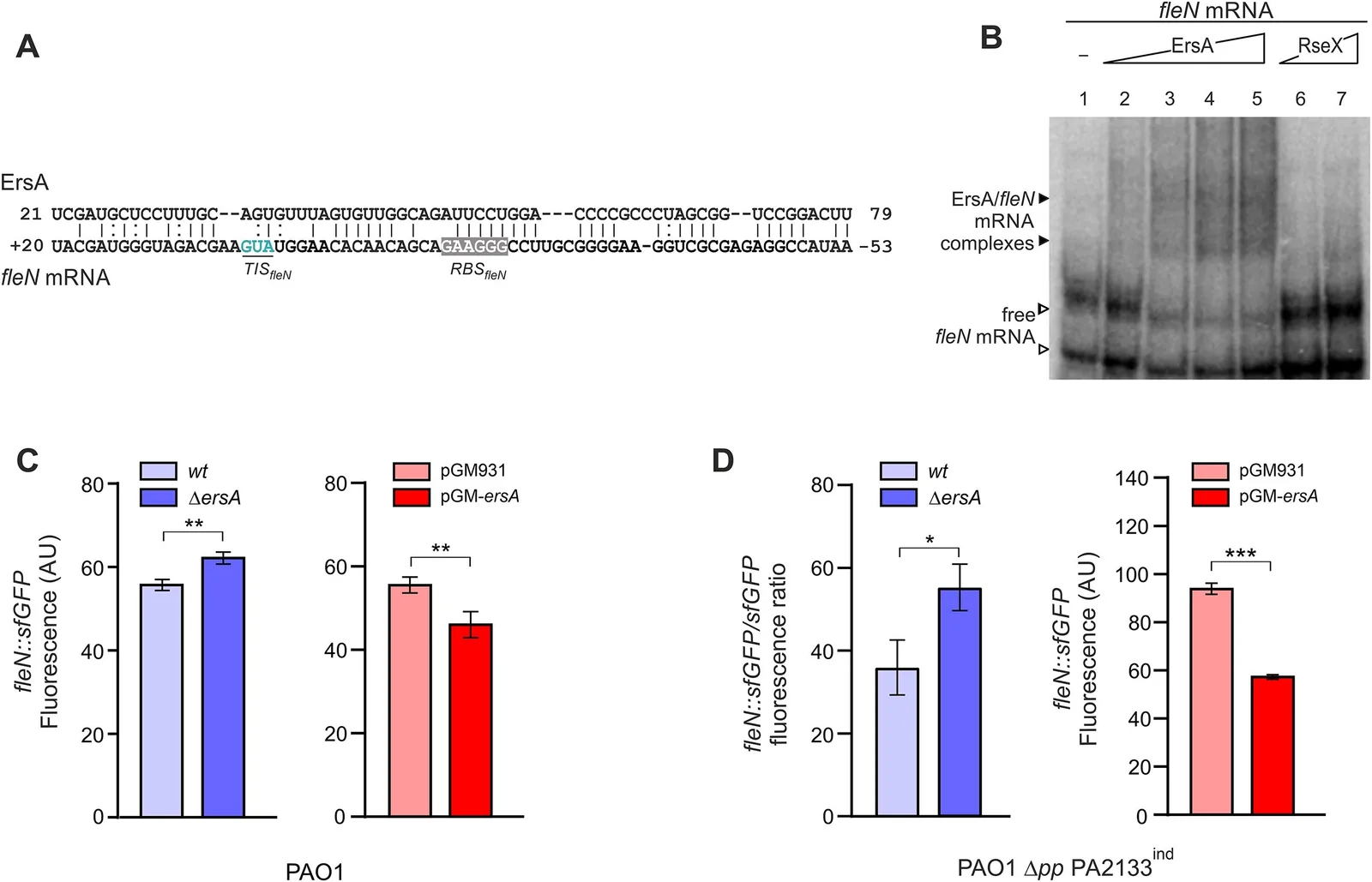
Influence of ErsA on post-transcriptional regulation of *fleN*. (**A**) Base pairing between the *fleN* mRNA (coordinates +18 to −53 from the AUG translation start site) and ErsA (from nucleotides 19–79) predicted by the web tool TargetRNA. The RBS of *fleN* mRNA (*RBS_fleN_*) and the translation initiation site (*TIS_fleN_*) are indicated. (**B**) *In vitro* analysis of the interaction between ErsA and *fleN* mRNA by electrophoretic mobility shift. Increasing amounts of purified ErsA RNA (0, 0.004, 0.04, 0.12, 0.24 pmol; lanes 1–5) or, as a negative control, *E. coli* RseX RNA (0.25, 1.5 pmol; lanes 6–7) were incubated at 37°C for 20 min with 0.04 pmol of radiolabeled *fleN* mRNA, loaded onto a native 6% polyacrylamide gel. White and black arrowheads indicate free *fleN* mRNA and ErsA/*fleN* mRNA complexes, respectively. (**C**) Comparison of fluorescence resulting from the *P_LtetO-1_*-driven *fleN::sfGFP* translational fusion of pBBR1-*fleN::sfGFP* in PAO1 wt versus PAO1 Δ*ersA*, and in PAO1 wt carrying pGM931 versus pGM-*ersA*. Data are expressed as the mean (*n* = 4) ± SD. (**D**) Comparison of fluorescence from pBBR1-*fleN::sfGFP* normalized to that from pBBR1-*sfGFP* in PAO1 Δ*pp* PA2133^ind^ versus PAO1 Δ*pp* PA2133^ind^ Δ*ersA*, and in PAO1 Δ*pp* PA2133^ind^ carrying pGM931 versus pGM-*ersA*. Data are expressed as the mean (*n* = 3) ± SD. Statistical analysis has been performed using Welch’s *t*-test (*P-value <.05; **P-value <.01; ***P-value <.001).

Following an approach described previously [37], we compared c-di-GMP levels in planktonic cells grown in shaken liquid medium (LSha) with cells in a colony biofilm (CBio) on the surface of an agar medium, a form of growth that reproduces most biofilm-associated traits [48]. Regardless of growth conditions, the lack of ErsA resulted in a 50%–70% increase in reporter levels (Fig. 4A). Collectively, these results suggested that ErsA functions as a negative regulator of c-di-GMP levels in both LSha and CBio. To validate this hypothesis, we directly quantified intracellular c-di-GMP levels via liquid chromatography-tandem mass spectrometry (LC-MS/MS) in PAO1 and PAO1 Δ*ersA* strains. Contrary to the reporter assay data, LC-MS/MS analysis revealed no significant differences in global c-di-GMP content between PAO1 and PAO1 Δ*ersA* strains (Fig. 4B). To address this discrepancy, we hypothesized that ErsA modulates the *cdrA* promoter post-transcriptionally by targeting specific regulatory factors, rather than by modulating global c-di-GMP levels. Control of the *cdrA* promoter relies on the coordinated activity of the c-di-GMP-responsive transcription factor FleQ and the protein FleN [22]. We focused our investigation on FleN, as our prior TargetRNA *in silico* analysis predicted *fleN*—alongside *algC* and *mlaA* mRNA—as a direct post-transcriptional target of ErsA. TargetRNA suggested an interaction between ErsA, from nt 19 to 79 spanning the unstructured region downstream of stem and loop SL1 and SL2, and the *fleN* mRNA from nucleotide –53 to +18 from the AUG translation start codon (Fig. 5A). This interaction was validated *in vitro* with purified components (Fig. 5B). Because the extended ErsA/*fleN* mRNA interaction encompassed *fleN* mRNA RBS and AUG start codon, we hypothesized that ErsA negatively regulates *fleN*. To evaluate this, we generated the reporter fusion plasmid pBBR1-*fleN::sfGFP* and transformed it into the PAO1 and PAO1 Δ*ersA* strains. As shown in Fig. 5C, the absence of ErsA resulted in higher reporter activity, consistent with a negative regulatory role of ErsA on *fleN* translation. Conversely, overexpression of ErsA via pGM-*ersA* reduced reporter activity compared to the empty vector control (Fig. 5C). Collectively, these results demonstrate that ErsA negatively regulates *fleN* translation. This regulatory effect provides an explanation for the elevated activity of the c-di-GMP-responsive pCdrA::*gfp*^C^ reporter in the PAO1 Δ*ersA* mutant, which occurs independently of alterations in global c-di-GMP levels (Fig. 4A and B).

Because c-di-GMP counteracts ErsA-mediated regulation of *algC* and *mlaA*, we investigated whether low c-di-GMP levels promote ErsA-dependent repression of *fleN*. To address this, we transformed the PAO1 Δ*pp* PA2133^ind^ strain and its corresponding Δ*ersA* mutant with the reporter plasmid pBBR1-*fleN::sfGFP*. In addition, PAO1 Δ*pp* PA2133^ind^ strain was co-transformed with pBBR1-*fleN::sfGFP* and either pGM-*ersA* or the empty vector control pGM931. As shown in Fig. 5D, both deletion and overexpression of ErsA resulted in consistent yet more pronounced effects in the low c-di-GMP PAO1 Δ*pp* PA2133^ind^ strain compared to PAO1 (Fig. 5C). These results suggest that ErsA regulation of *fleN* is also sensitive to c-di-GMP levels.

## Discussion

A distinctive feature of ErsA is that it operates under the positive control of the envelope stress-responsive sigma factor σ^22^ [28], the functional homolog of *E. coli* σ^E^ [49]. In *P. aeruginosa*, σ^22^ was initially identified not in the context of the envelope stress response, but for its essential role in the expression of the *alg* genes involved in the production of the biofilm matrix exopolysaccharide alginate [50]. Later, σ^22^ was shown to have a large regulon of about 290 genes associated with the response to cell wall stress, including genes for outer envelope biogenesis and remodeling [51]. Furthermore, members of the *P. aeruginosa* σ^22^ regulon have been shown to affect the stress response system itself and to play a role in envelope homeostasis [52]. The effects of mutations in some genes belonging to the σ^22^ regulon provided evidence that the σ^22^ stimulon for cell envelope homeostasis overlaps with biofilm control mechanisms [52]. In *P. aeruginosa*, the link between envelope stress and biofilm control is also supported by surface-sensing pathways. The Wsp chemosensory-like signal transduction pathway senses surfaces and promotes biofilm formation [12, 53]. Surface attachment is thought to induce local envelope stress characterized by the accumulation of misfolded or aberrant periplasmic and inner membrane proteins [53]. This structural perturbation triggers a downstream phosphorylation cascade that activates the DGC WspR. The resulting elevation in intracellular c-di-GMP levels subsequently reinforces surface adherence and drives biofilm maturation.

Biofilm formation thus results from the convergence of stress signals routed through both the Wsp and σ^22^ pathways. This interaction is reflected in the regulation of the alginate biosynthesis genes, including *alg*C and the *alg* operon, which are activated by σ^22^ and by a Wsp-dependent increase in c-di-GMP levels [19–21, 26, 27]. Nevertheless, the extent of crosstalk between the Wsp and σ^22^ pathways, their activating signals, and the functions that they regulate remain only partially defined.

The biofilm-promoting roles of σ^22^ and c-di-GMP appeared incongruent with the σ^22^-stimulated repression of ErsA on AlgC, an enzyme central for supplying precursors for biofilm exopolysaccharide biosynthesis. To disentangle this, we assessed whether c-di-GMP could function to offset the ErsA-mediated repression of AlgC. Our *in vivo* data indicate that c-di-GMP levels influence ErsA-mediated control of AlgC, not by affecting ErsA abundance, but by alleviating its post-transcriptional repression at high intracellular concentrations. Our *in vitro* results indicate that c-di-GMP can interfere with the formation of the ErsA/*algC* mRNA complex, strongly supporting the post-transcriptional effect observed *in vivo*.

Beyond this post-transcriptional effect on ErsA function, we observed that high c-di-GMP levels correlate with an increase in *algC* mRNA. This suggests that c-di-GMP enhances AlgC expression through coordinated regulation at both the transcriptional and post-transcriptional levels during biofilm formation. This regulatory architecture is expected to enable sustained production of exopolysaccharide precursors during biofilm formation, even under σ^22^-mediated stress conditions that would otherwise elevate ErsA levels.

We identified, as a novel ErsA target, the *mlaA* gene, which encodes MlaA, an OM lipoprotein involved in the removal of mislocalized glycerophospholipids at the outer leaflet of the OM, thus maintaining lipid asymmetry [29, 30]. Our findings reveal a comparable c-di-GMP-dependent regulatory framework, albeit with distinct features. We show that *mlaA* mRNA is similarly subjected to ErsA-mediated repression, which is alleviated at high c-di-GMP levels. Like *algC*, we observed that high levels of c-di-GMP give rise to increased abundance of *mlaA* mRNA. However, ErsA appears to play an additional role in this context, as its overexpression further enhances *mlaA* transcript levels, suggesting a positive effect on *mlaA* mRNA stability and/or transcription. A key distinction from *algC* is that high c-di-GMP levels actively enhance *mlaA* translation. This regulatory module further highlights the ErsA bridging activity between the σ^22^ pathway and c-di-GMP signaling: the combination of high c-di-GMP levels and an active σ^22^ pathway, which stimulates ErsA transcription, maximizes the expression of MlaA. The lack of MlaA has been associated with a decrease in the secretion of rhamnolipids, motility, and biofilm formation, as well as an increase in susceptibility to fluoroquinolones, bacterial virulence, and the innate immune response [54, 55]. We propose that the MlaA upregulation under high c-di-GMP levels may contribute to chronic infection by favoring biofilm formation and, at the same time, reducing bacterial virulence and the innate immune response. Under these conditions, ErsA-mediated repression of MlaA would counteract this lifestyle. Thus, relief of ErsA-mediated repression of MlaA by high levels of c-di-GMP may be a mechanism that perpetuates chronic infection. Moreover, the dependence of ErsA-mediated MlaA repression on c-di-GMP levels may also be implicated in the transition from chronic to acute infection. If host conditions result in a drop in c-di-GMP levels, increased repression of *mlaA* mRNA by ErsA could contribute to the outcome of higher bacterial virulence and innate immune response.

By identifying the *fleN* gene as an ErsA target, we further expanded the knowledge of ErsA as a major regulator of biofilm matrix biosynthesis. FleN and the transcription factor FleQ drive the c-di-GMP-dependent transcriptional switch between biofilm-promoting and flagellar genes [22]. Under high c-di-GMP conditions, the *pel, psl*, and *cdrAB* operons are upregulated while flagellar genes are repressed; conversely, low c-di-GMP levels reverse this expression profile. FleN plays a positive role in the induction of biofilm-promoting genes, as inactivation of the *fleN* gene results in reduced expression of *pel, psl*, and *cdrAB* operons [56]. Our results indicate a negative role of ErsA on *fleN* mRNA translation that mirrors that of *algC* mRNA. Thus, ErsA serves as a c-di-GMP-sensitive tuner of biofilm matrix biosynthesis acting at both the metabolic (*algC*) and transcriptional (*fleN*) levels. We speculate that ErsA can play a role in modulating the transition between planktonic and biofilm modes of growth, as the FleQ/FleN pair is a pivotal regulatory element of this process [22].

A defining feature enabling ErsA to act as a c-di-GMP-dependent fine-tuner is the sensitivity of its post-transcriptional regulatory activity to intracellular c-di-GMP concentrations. Several nonmutually exclusive mechanisms may underlie this c-di-GMP responsiveness: (i) fluctuations in c-di-GMP levels may modulate Hfq expression through intermediate regulatory factors [57, 58], thereby indirectly affecting the regulation of ErsA target mRNAs; (ii) specific c-di-GMP effectors may directly influence the chaperone activity of Hfq; (iii) c-di-GMP-responsive RNA-binding proteins could sequester ErsA and/or its target transcripts; and (iv) c-di-GMP may directly modulate the interaction between ErsA and its target mRNAs. Notably, the c-di-GMP sensitivity of ErsA-mediated repression appears to be target-specific, as it is not observed for *oprD* mRNA. This observation argues against mechanisms involving global modulation of Hfq activity or sequestration of ErsA. Furthermore, *in vitro* evidence demonstrating that c-di-GMP interferes with the interaction between ErsA and *algC* mRNA supports a model in which direct binding of c-di-GMP contributes to the alleviation of ErsA-mediated repression *in vivo*. The differential responsiveness of ErsA targets to c-di-GMP may reflect differences in the architecture and stability of the underlying RNA-RNA interactions. While ErsA is predicted to form relatively extensive base-pairing interactions with *algC, mlaA*, and *fleN* mRNAs, its interaction with *oprD* mRNA is limited to a perfectly complementary 10-nt duplex involving the *oprD* RBS and nucleotides immediately downstream of ErsA SL1 [32]. Such a short, highly complementary interaction may be less susceptible to perturbation by c-di-GMP, thereby accounting for the absence of an observable effect on ErsA-mediated regulation of *oprD* mRNA.

**Figure 6.**
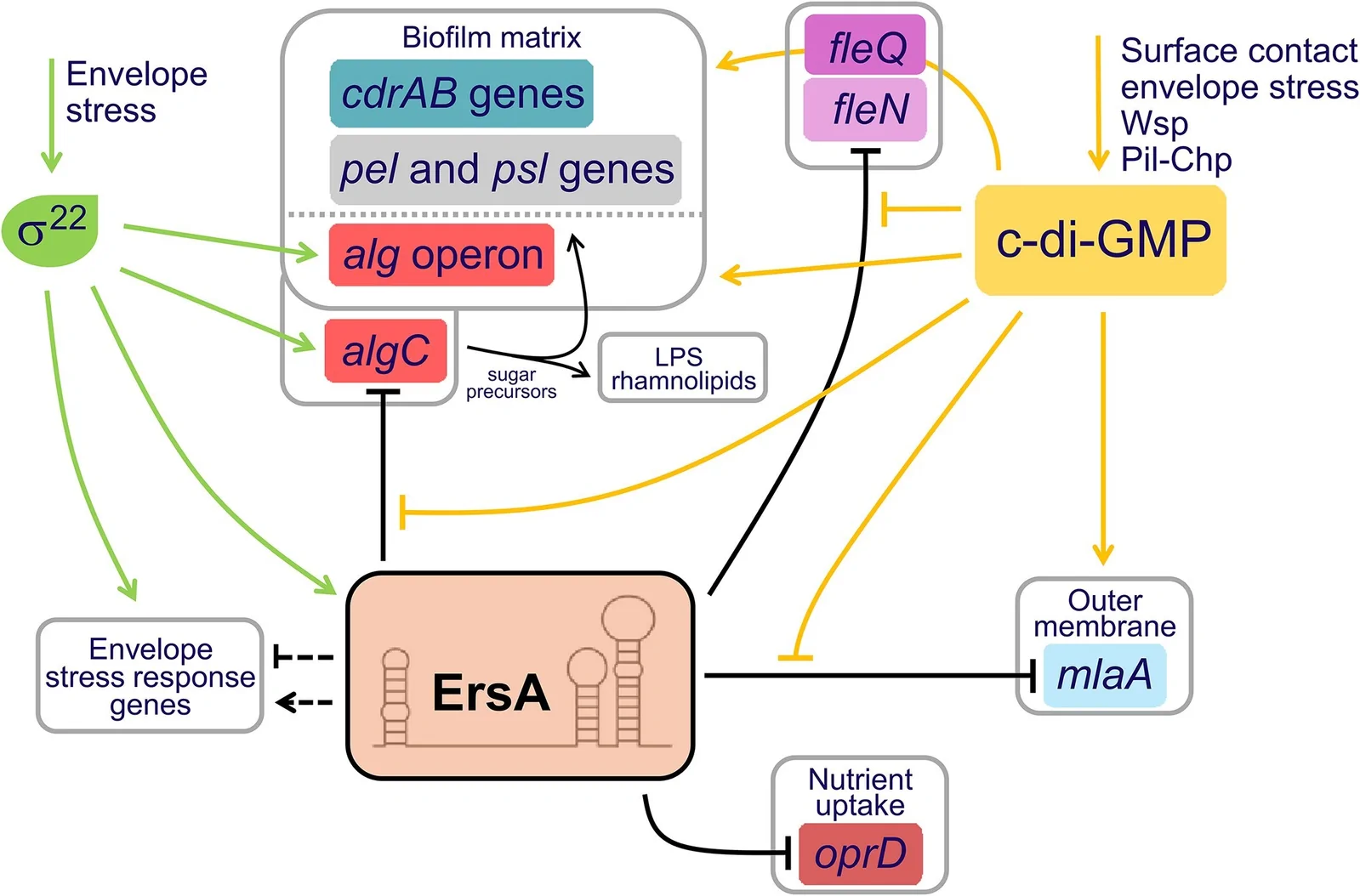
Schematic representation of the regulatory links that ErsA forms between the σ^22^ pathway and the c-di-GMP signaling networks. ErsA is depicted as a regulatory element linking inputs from the σ^22^ pathway with the c-di-GMP signaling triggered by activated Wsp and Pil-Chp systems. Biofilm matrix formation appears to emerge from overlapping signals routed through both the σ^22^ (green arrows) and Wsp (orange arrows) pathways. The alginate production genes in the *alg* operon are under positive control by σ^22^ and, at the same time, by high c-di-GMP levels. The same occurs for *algC*, which encodes a central enzyme in the production of sugar precursors for biofilm matrix exopolysaccharides. The other biofilm matrix exopolysaccharides Pel/Psl and the CdrAB adhesins are positively controlled by c-di-GMP with the involvement of the transcription factor FleQ and its regulator FleN. For biofilm matrix formation, a post-transcriptional bridge is established between the σ^22^ pathway and c-di-GMP signaling via ErsA. Both *algC* and *fleN* genes are subjected to ErsA-mediated repression (black blunt-end arrow), which is alleviated at high c-di-GMP levels (orange blunt-end arrow). Thus, ErsA serves as a c-di-GMP-sensitive tuner of biofilm matrix biosynthesis acting at both the metabolic (*algC*) and transcriptional (*fleN*) levels. The *mlaA* gene encoding an envelope homeostasis lipoprotein is positively controlled by c-di-GMP and is under a similar c-di-GMP-sensitive post-transcriptional regulation by ErsA. In contrast to *algC, fleN*, and *mlaA*, ErsA-mediated repression of *oprD* is c-di-GMP-insensitive. Finally, the potential role of ErsA in the regulation of envelope stress response genes (dashed arrow and blunt-end arrow) is also indicated.

The results of this study extend our early model on ErsA regulation [28]. We proposed that the incoherent regulatory loop involving σ^22^ and ErsA played a role in fine-tuning AlgC expression. Furthermore, by targeting genes in the σ^22^ regulon as a repressing arm, we hypothesized that ErsA might play a role similar to that of the σ^E^-dependent sRNAs MicA, RybB, and MicL in *E. coli* for the regulation of cell envelope homeostasis [59, 60]. Here, we present a model wherein ErsA serves as a central, c-di-GMP-responsive regulatory hub, linking envelope stress responses to c-di-GMP cascades to fine-tune key extracytoplasmic functions of biofilm matrix biosynthesis and OM homeostasis (Fig. 6). Our study reveals a novel, sophisticated multilayered control mechanism by which a sRNA integrates signals from envelope stress and c-di-GMP signaling to orchestrate key physiological and virulence-related decisions. This highlights a new interconnection between sRNAs and c-di-GMP [61]. Far from being a static repressor, ErsA is revealed to be a dynamic regulatory hub that enables the bacterium to finely tune gene expression in response to fluctuating environmental cues, a critical capability for the pathogenic lifestyle. Consistent with this role, we previously showed that the lack of ErsA leads to the formation of an immature biofilm [62]. The ErsA bridging activity may be key in fine-tuning the c-di-GMP signaling at one or more steps of biofilm development [12]. Importantly, this regulatory interplay may be modulated by environmental conditions such as oxygen availability, since c-di-GMP synthesis is regulated in an oxygen-dependent manner via SadC [63], and ErsA influences anaerobic metabolism through post-transcriptional modulation of the master regulator Anr [64]. Based on the observation that post-transcriptional regulation of certain ErsA targets (*algC, mlaA*, and *fleN*) is alleviated under elevated c-di-GMP levels, whereas others (such as *oprD*) remain unaffected, we propose that c-di-GMP responsiveness is target-dependent within the ErsA regulon, enabling functional diversification of ErsA-mediated regulatory outcomes.

In summary, our results highlight an underappreciated role of sRNAs as integrators of bacterial signaling pathways, bridging stress responses and second messenger signaling. This work provides a valuable foundation for future studies exploring how sRNA-mediated post-transcriptional regulation shapes bacterial pathogenesis and adaptation.

## Supplementary Material

gkag930_Supplemental_File

## Data Availability

The data underlying this article are available in the article and in its online supplementary material.
