# Supplementary material for: Linking the second messenger cyclic di-GMP signaling with the regulatory activity of the small RNA ErsA in Pseudomonas aeruginosa
Source: Nucleic Acids Res. 2026 Sep 25;54(18):gkag930. doi: 10.1093/nar/gkag930 (PMC13613067; doi:10.1093/nar/gkag930)
Supplement: Supplementary file 1 [file gkag930_supplemental_file.pdf]

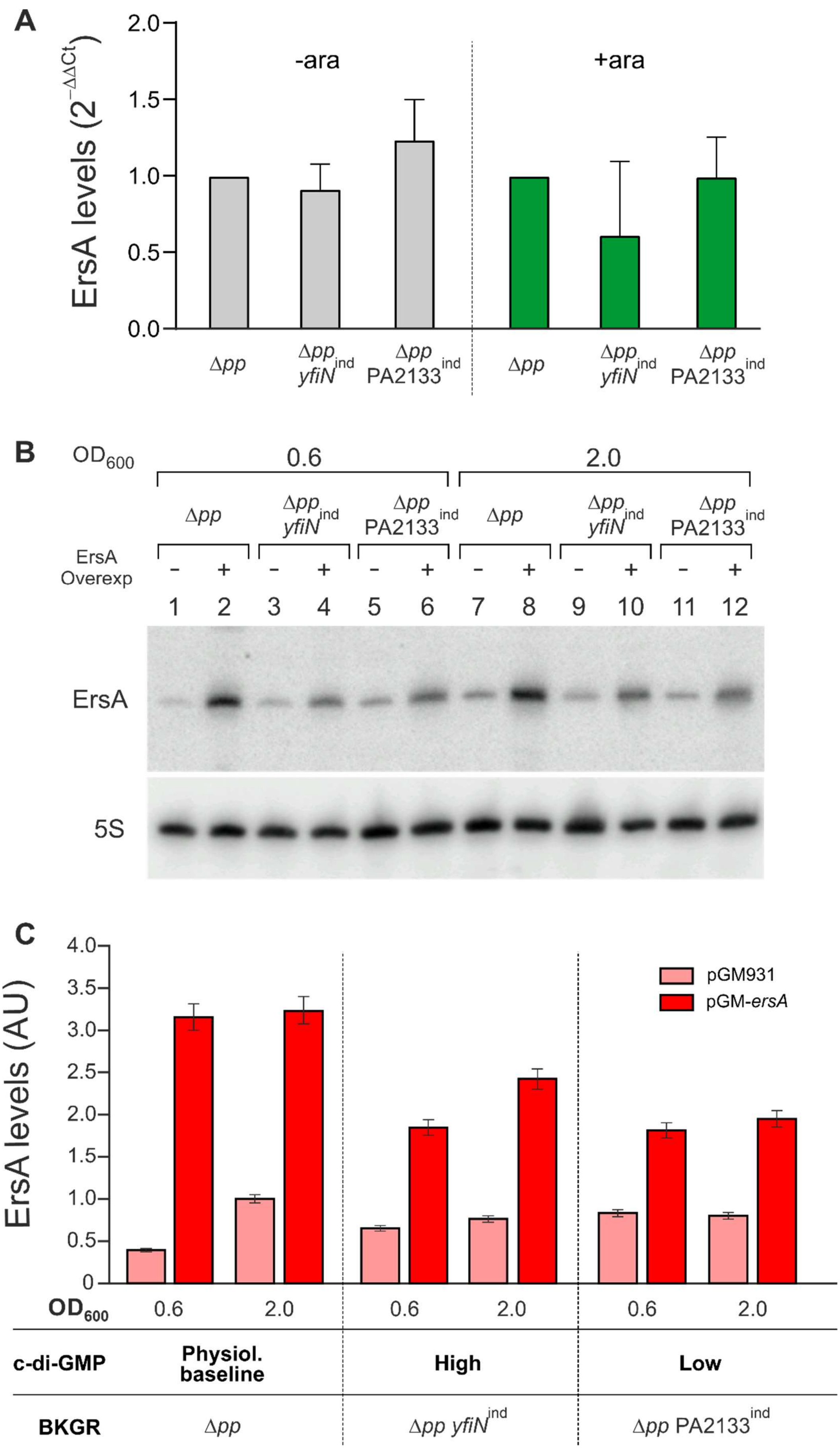

A
ErsA levels ($2^{-\Delta\Delta Ct}$)
2.0
1.5
1.0
0.5
0.0
-ara
+ara
Δpp
Δpp yfiN ind
Δpp PA2133 ind
Δpp
Δpp yfiN ind
Δpp PA2133 ind
B
OD600
0.6
2.0
Δpp
Δpp yfiN ind
Δpp PA2133 ind
Δpp
Δpp yfiN ind
Δpp PA2133 ind
ErsA Overexp
- + - + - + - + - + - +
1 2 3 4 5 6 7 8 9 10 11 12
ErsA
5S
C
ErsA levels (AU)
4.0
3.5
3.0
2.5
2.0
1.5
1.0
0.5
0
pGM931
pGM-ersA
OD600
0.6
2.0
0.6
2.0
0.6
2.0
c-di-GMP
Physiol. baseline
High
Low
BKGR
Δpp
Δpp yfiN ind
Δpp PA2133 ind

Figure S1. Evaluation of ErsA abundance and overexpression at different intracellular c-di-GMP levels. (**A**) Comparison of ErsA levels in PAO1 Δ*pp*, PAO1 Δ*pp yfiN*^{ind,} and PAO1 Δ*pp* PA2133^{ind} determined by RT-qPCR and displayed graphically as $2^{-\Delta\Delta Ct}$ values relative to PAO1 Δ*pp*. Cultures of these strains were grown at 37°C in LB medium with and without arabinose, sampled at an OD_{600} of 2, and processed for total RNA extraction. RNA was extracted from three independent biological replicates, and RT-qPCR reactions were performed in triplicate for each biological replicate. (**B**) Northern blot analysis of ErsA levels in PAO1 Δ*pp,* Δ*pp yfiN*^{ind,} and Δ*pp* PA2133^{ind}, harboring either the empty vector pGM931 (ErsA overexp: -) or the vector pGM-*ersA* (ErsA overexp: +), to evaluate endogenous ErsA levels or ErsA overexpression, respectively*.* These strains were grown at 37°C in LB medium with carbenicillin and arabinose, sampled at the indicated $OD_{600,}$ and processed for total RNA extraction. (**C**) Intensities of ErsA bands in (**B**) were quantified and normalized to those of 5S RNA in the same lane. Values are expressed in arbitrary units (AU) and represent the mean ± SD of three biological replicates. Statistical analysis was performed by one-way ANOVA with *post-hoc* Tukey's honestly significant difference test. No statistically significant differences were detected (*p*-value>0.05).

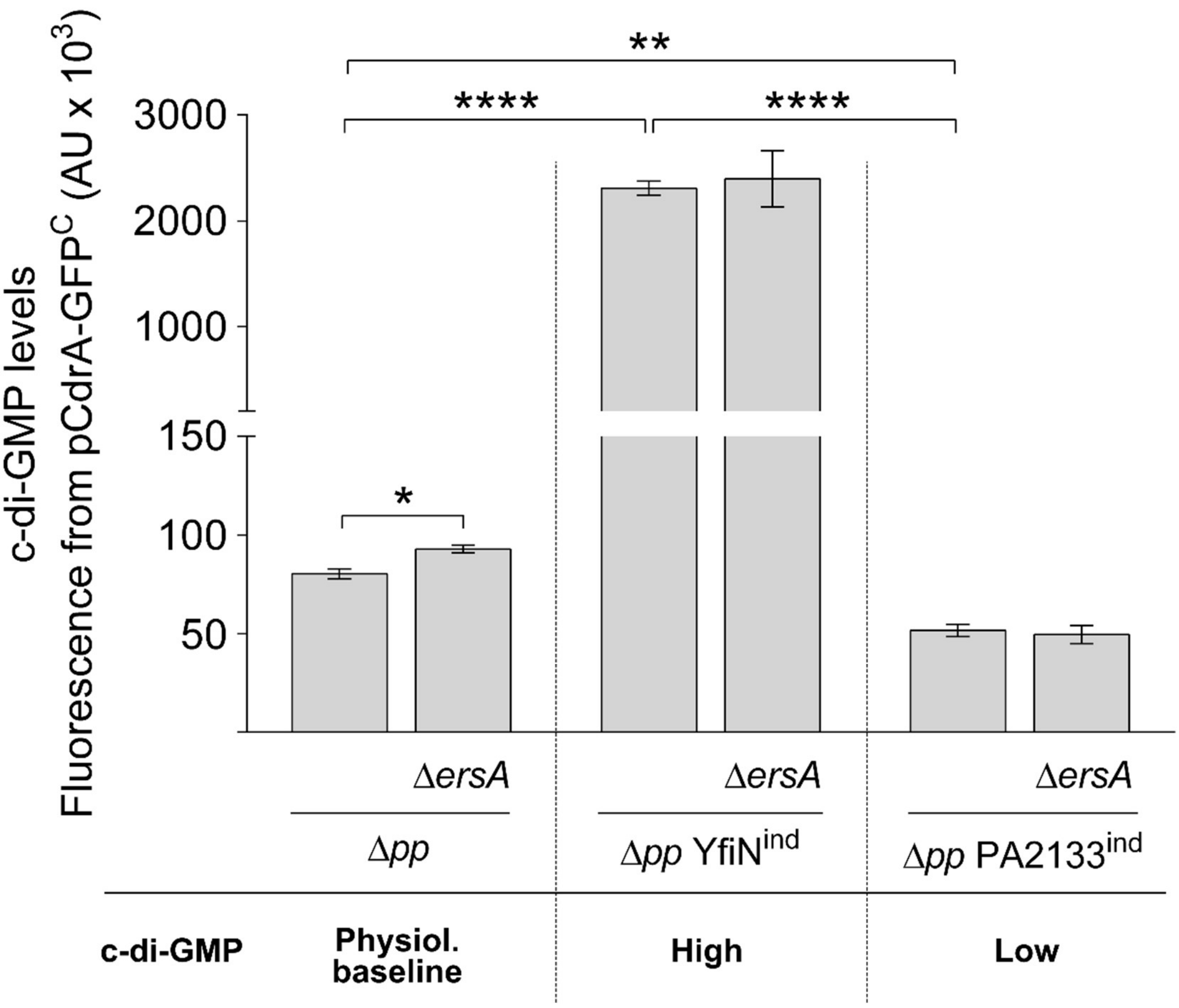

Figure S2. Evaluation of the influence of ErsA on the dysregulation of intracellular c-di-GMP levels. PAO1 Δ*pp*, PAO1 Δ*pp yfiN*^{ind}, PAO1 Δ*pp* PA2133^{ind,} and their corresponding Δ*ersA* mutans were transformed with the plasmid pCdrA::*gfp*^{C}. Bars represent the quantitative evaluation of fluorescence levels produced by cultures of these strains grown until OD_{600} of 2 at 37°C in liquid LB medium with arabinose. Values are expressed in arbitrary units (AU) and represent the mean ± SD of three biological replicates. Statistical significance was calculated by one-way ANOVA with *post-hoc* Tukey's honestly significant difference test (* *p*-value<0.05; ** *p*-value<0.01; **** *p*-value<0.0001).

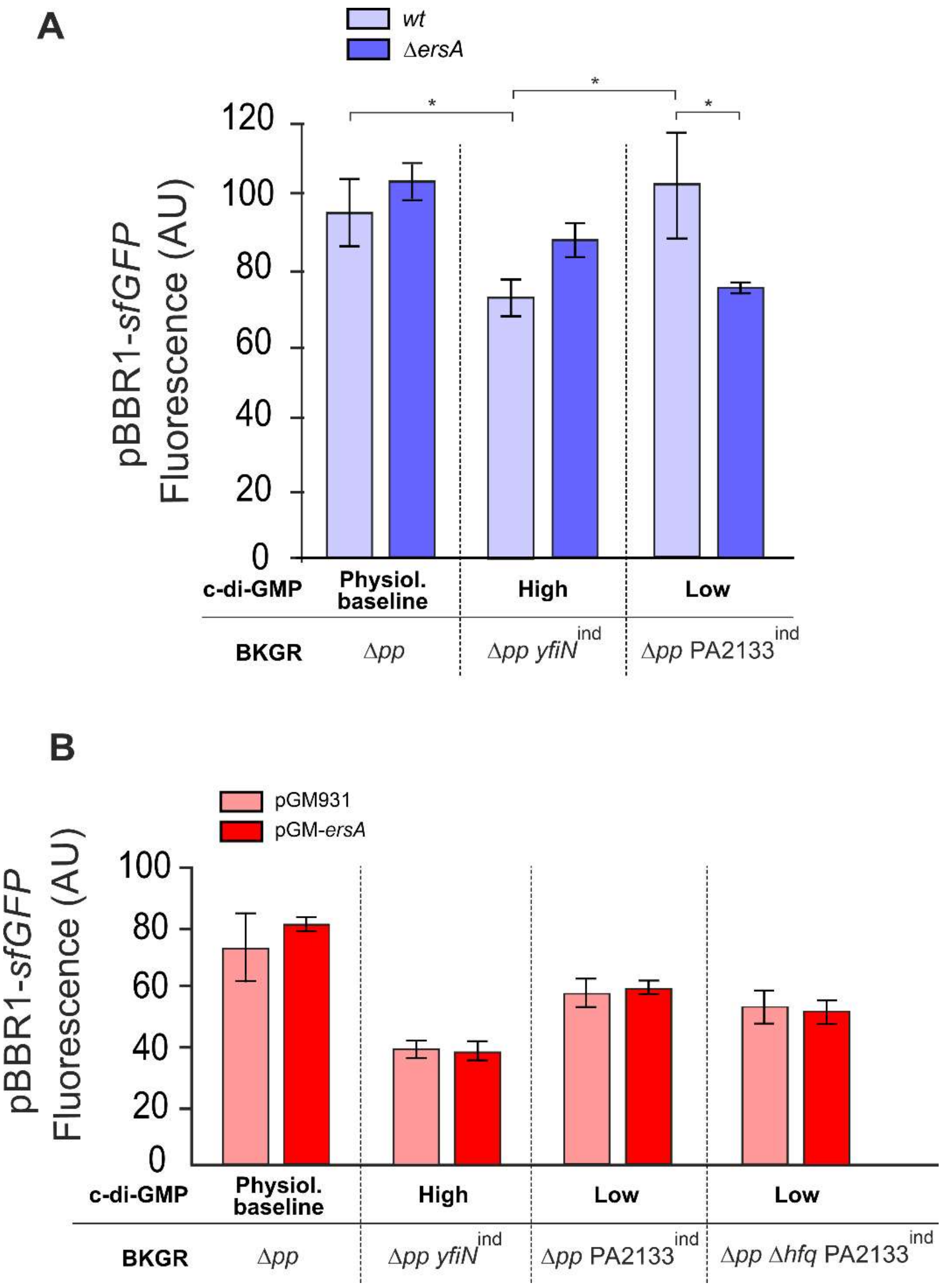

Figure S3. Influence of the intracellular c-di-GMP levels on the expression of the *sfGFP* gene *per se*. (**A**) PAO1 Δ*pp*, PAO1 Δ*pp yfiN*^{ind}, PAO1 Δ*pp* PA2133^{ind}, and their corresponding Δ*ersA* mutants were transformed with the plasmid pBBR1-sf*GFP*. Bars represent the quantitative evaluation of fluorescence levels produced by cultures of these strains grown until OD_{600} of 2 at 37°C in liquid LB medium with arabinose. Intrinsic fluorescence was negligible in PAO1 Δ*pp*, PAO1 Δ*pp yfiN*^{ind}, PAO1 Δ*pp* PA2133^{ind}, and their corresponding Δ*ersA* mutant strains lacking pBBR1-sf*GFP* plasmid. (**B**) PAO1 Δ*pp*, PAO1 Δ*pp yfiN*^{ind}, PAO1 Δ*pp* PA2133^{ind,} and PAO1 Δ*pp* Δ*hfq* PA2133^{ind} were transformed with pBBR1-sf*GFP* and with the empty vector pGM931 or pGM-*ersA*. Bars represent the quantitative evaluation of fluorescence levels produced by cultures of these strains grown until OD_{600} of 2 at 37°C in liquid LB medium with arabinose. Values are expressed in arbitrary units (AU) and represent the mean ± SD of three biological replicates. Statistical significance was calculated by one-way ANOVA with *post-hoc* Tukey's honestly significant difference test (* *p*-value<0.05).

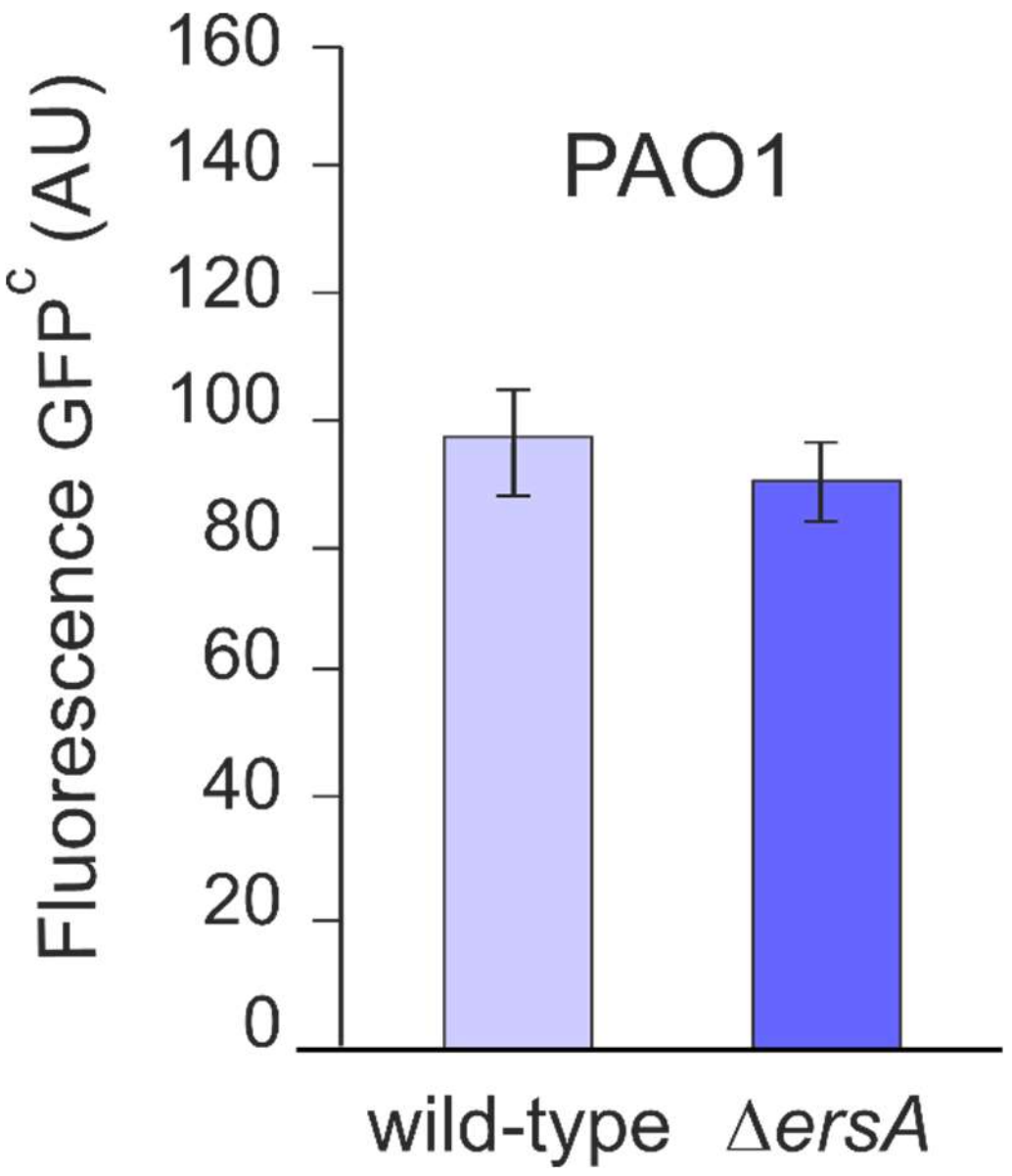

Figure S4. Expression of the *gfp*^{C} reporter gene in PAO1 and PAO1 Δ*ersA*. The plasmid pBBR1-*gfp*^{C}, equivalent to pCdrA::*gfp*^{C}, but with the *gfp*^{C} gene transcriptionally fused to the constitutive $P_{LtetO-1}$ promoter, was tested in the two genetic backgrounds. Bars represent the quantitative evaluation of fluorescence levels produced by cells grown until OD_{600} of 2 at 37°C in liquid LB medium with shaking (LSha). Values are expressed in arbitrary units (AU) and represent the mean ± SD of three biological replicates. Statistical analysis was performed using Welch's *t*-test. No statistically significant differences were detected (*p*-value>0.05).

Table S1. Strains and plasmids

Strains or plasmids	Genotype/Relevant features	Construction	Reference
Pseudomonas aeruginosa			
PAO1	PAO1 wild-type		(1)
PAO1 *hfq*⁻	PAO1 Hfq::*aadA*, Sm/Sp^R		(2)
PAO1 Δ*ersA*	PAO1 Δ*ersA*, (markerless)		(3)
PAO1 Δ*pp*	PAO1 Δ*pelABCD* Δ*pslABCD*, (markerless)		(5)
PAO1 Δ*pp* Δ*ersA*	PAO1 Δ*pelABCD* Δ*pslABCD* Δ*ersA*, (markerless)		This study
PAO1 Δ*pp* *yfiN*^{ind}	PAO1 Δ*pelABCD* Δ*pslABCD::*P_{BAD} *-YfiN*^{ind} *(single-copy chromosomal integration),* Tc^R		(4)
PAO1 Δ*pp* Δ*ersA* *yfiN*^{ind}	Δ*pelABCD* Δ*pslABCD* Δ*ersA::*P_{BAD}*-YfiN*^{ind} *(single-copy chromosomal integration),* Tc^R		This study
PAO1 Δ*pp* PA2133^{ind}	Δ*pelABCD*, Δ*pslABCD::*P_{BAD}*-PA2133*^{ind} *(single-copy chromosomal integration),* Tc^R		(4)
PAO1 Δ*pp* Δ*ersA* PA2133^{ind}	markerless Δ*pelABCD* Δ*pslABCD* Δ*ersA::* P_{BAD}*-PA2133*^{ind} *(single-copy chromosomal integration),* Tc^R		This study
PAO1 Δ*pp* Δ*hfq* PA2133^{ind}	Δ*pelABCD* Δ*pslABCD* Δ*hfq::*P_{BAD}*-PA2133*^{ind} *(single-copy chromosomal integration),* Tc^R		This study
Escherichia coli			
TOP10	*mcrA Δ(mrr-hsdRMS-mcrBC) φ80lacZDM15 ΔlacX74 deoR recA1 araD139 Δ(ara-leu)7697 galU galK rpsL endA1 nupG*		Invitrogen
C1a	*E. coli C*, prototrophic		(5)
CC118 *λpir*	*Δ (ara-leu), araD, ΔlacX174, galE, galK, phoA, thi1, rpsE, rpoB, argE (Am), recA1, lysogenic λpir*		(6)
HB101(pRK600)	*recA thi pro leu hsd M* $^+$ Sm^R		(7)
Plasmids			
pGM931	pHERD20T derivative, araC/P_{BAD} - t_{Ω}, Ap^R		(8, 9)
pGM-*ersA*	pGM931 derivative, expressing *ersA* under P_{BAD}, Ap^R		(3)
pGM-*ersA*mut	pGM931 derivative, expressing mutant *ersA* (30-32 CTT→GGA), under P_{BAD}, Ap^R	Contains *ersA* mutated at position 30-32 (CTT→GGA), generated using oligos 12/13	This study
pCdrA::*gfp*^{C}	It carries the c-di-GMP-responsive *cdrA* promoter transcriptionally fused to the green fluorescence protein *gfp*^{C} gene		(10)

pXG10-SF	sfGFP reporter plasmid; *lacZ*::*sfGFP* under $P_{LtetO-1}$, Cm^R		(11)
pXG10-*gfp*^{C}	pXG10-SF and pCdrA::*gfp*^{C} derivative; $P_{LtetO-1}$ →*lacZ::*GFP^C, Cm^R	contains *gfp*^{C} PCR product using oligo 11/12	This study
pXG10-*mlaA::sfGFP*	pXG10-SF derivative; $P_{LtetO-1}$ →*mlaA::sfGFP*, Cm^R	contains PCR product using oligo 13/14	This study
pXG10-*oprD::sfGFP*	pXG10-SF derivative; $P_{LtetO-1}$ →*oprD::sfGFP*, Cm^R	contains PCR product using oligo 15/16	This study
pXG10-*fleN::sfGFP*	pXG10-SF derivative; $P_{LtetO-1}$ →*fleN::sfGFP*, Cm^R	contains PCR product using oligo 17/18	This study
pBBR1-MCS5	REP, lacZ alpha, Gm^R		(12)
pBBR1-*sfGFP*	sfGFP reporter plasmid; $P_{LtetO-1}$→*sfGFP*, Gm^R		(3)
pBBR1-*gfp*^{C}	GFP^C reporter plasmid; $P_{LtetO-1}$ →*lacZ::gfp*^{C}, Gm^R	contains PCR product using oligo 19/20	This study
pBBR1-*algC::sfGFP*	pBBR1-MCS5 and pXG10-*algC::sfGFP* derivative, $P_{LtetO-1}$ →*algC::sfGFP*, Gm^R		(3)
pBBR1-*mlaA::sfGFP*	pBBR1-MCS5 and pXG10-*mlaA::sfGFP* derivative, $P_{LtetO-1}$ →*mlaA::sfGFP*, Gm^R	contains PCR product using oligo 19/20	This study
pBBR1-*oprD::sfGFP*	pBBR1-MCS5 and pXG10-*oprD::sfGFP* derivative, $P_{LtetO-1}$ →*oprD::sfGFP*, Gm^R	contains PCR product using oligo 19/20	This study
pBBR1-*fleN::sfGFP*	pBBR1-MCS5 and pXG10-*fleN::sfGFP* derivative, $P_{LtetO-1}$ →*fleN::sfGFP*, Gm^R	contains PCR product using oligo 19/20	This study
pSEVApao1-Δ*ersA*	pSEVA612S derivative, Ap^R		(3)
pSEVApa14-Δ*hfq*	pSEVA612S derivative, Ap^R		(13)
pSW-1	oriRK2, *xylS*; *Pm*→I-SceI, Ap^R		(14)

Table S2. Oligonucleotides

Oligo #	Oligo Name	Sequence (5' → 3') [a]	Utilization and references
1	SPA0122	CCCGAGCTTCGTATGGGG	Northern-blot probe for ErsA (15)
2	5'BIOTIN-SPA122	[BIO]CCCGAGCTTCGTATGGGG	Biotinylated Northern-blot probe for ErsA
3	PA5SRNA02	GGAGACCCCACACTACCATCGGCGATG	Northern-blot probe for 5S (15)
4	T7_5'SPA0122_f	**CTAATACGACTCACTATAGGG**CGAATGGCTTCTTGAGCC	Amplification of ErsA in fusion with RNA polymerase T7 promoter for *in vitro* transcription (3)
5	3'SPA0122_r	AAAAAAAACCCCGAGCTTCGTATGGGGAG	
6	T7_-75ATG_AlgC_f	**CTAATACGACTCACTATAGGG**CAGAACACCGA	Amplification of the 5' UTR and beginning of *algC* in fusion with RNA polymerase T7 promoter for *in vitro* transcription (15)
7	+83ATG_AlgC_r	CGGTGAGGGTATCGCCTACCACGC	
8	T7_-79ATG_PA2800_f	**CTAATACGACTCACTATAGGG**ATCGATGCGAGCCTATAT	Amplification of the 5' UTR and beginning of *mlaA* in fusion with RNA polymerase T7 promoter for *in vitro* transcription
8	+106ATG_PA2800_r	GGTCTTCTTCACTGGCCGCCAGGGA	
10	T7_ -112ATG_FleN_f	ctaatacgactcactatagggGCGGTGACGGAAGCGAAGGT	Amplification of the 5' UTR and beginning of *fleN* in fusion with RNA polymerase T7 promoter for *in vitro* transcription
11	+90ATG_FleN_f	GTTCACCGACACATTGGTCTTGCCGA	
12	T7_RseX_f	**CTAATACGACTCACTATAGGG**TTTTTATTATTCTGTGTCATGATG	Amplification of RseX in fusion with RNA polymerase T7 promoter for *in vitro* transcription (3)
13	RseX_r	TAAAAAAAAGCCGGCATCAT	
14	NcoI_5'ErsACCTmutGGA_for	GAccatggACGAATGGCTTCTTGAGCCCTTCGATGCTGGATTGCA	Amplification of *ersA* with 30-32 CTT→GGA mutation; *Nco*I/*Pst*I ends for cloning into pGM931
15	PstI_3'SPA0122_r	GAActgcagAAAAAAAACCCCGAGCTTCGTA	
16	GFPc_NheI_for	GTTTTgctagcCGTAAAGGAGAAGAAC	Amplification of *gfp*[C] (codon 2 to stop) from pCdrA::*gfp*[C]; *Nhe*I/*Xba*I ends for cloning into pXG10-SF
17	GFPc_XbaI_rev	GTTTtctagaTTATTTGTATAGTTC	
18	-79ATG_PA2800_sfGFP_NsiI _f	GTTTTATGCATATCGATGCGAGCCTATATACTGCGC	Amplification of *mlaA* region (-79 to +105 nt from *mlaA* start codon) with *Nsi*I/*Nhe*I ends for in-frame cloning with sfGFP into pXG10-SF
19	PA2800_+105ATG_NheI_r	GTTTTgctagcGTCTTCTTCACTGGCCG	
20	-78ATG_oprD_NsiI_f	GTTTTatgcatAGCGCCGACAAGAAGAAC	Amplification of *oprD* region (-78 to +90 from start codon) with *Nsi*I/*Nhe*I ends for in-frame cloning with sfGFP into pXG10-SF
21	oprD_+90ATG_NheI_r	GTTTTgctagcCTGATCGCTG ACGAATGC	
22	-112ATG_FleN_NsiI_f	GTTTTatgcatCGGGTGACGGAAGCGAAGGT	Amplification of *fleN* region (-112 to +90 from start codon) with *Nsi*I/*Nhe*I ends for in-frame cloning with sfGFP into pXG10-SF
23	FleN_+90ATG_NheI_r	GTTTTgctagcGTTCACCGACACATTGGTCTTGCCGA	

24	Ptet-O1_ClaI_f	GTTTTatcgatTCCCTATCAGTGATAGAG	Amplification from the $P_{LtetO-1}$ promoter to sfGFP stop codon (pXG10- derivative) with *Cla*I/*Xba*I ends for cloning into pBBR1-MCS5 (3)
25	sfGFP_TAA_XbaI_r	TGATGCCtctagaTTATTTGTAGAGCTC	
26	sfGFP_+96_r	TTGTGCCCATTAACATCACCATC	Reverse primer on *sfGFP* for verification of constructs (3)
27	pSEVA_f	TAAAACGACGGCCAGTATAGGG	Forward primer on pSEVA vector for verification of constructs
28	pSEVA_r	CAGCTATGACCATGATTACGCC	Reverse primer on pSEVA vector for verification of constructs
29	spa0122_300up	GATAAAGCGCACACAAAATCC	Forward primer on 300 bp upstream of the *ersA* gene for verification of deletion (3)
30	spa0122_300down	GCATCCATACCTCATACCACC	Reverse primer on 300 bp downstream of the *ersA* gene for verification of deletion (3)
31	TS1hfqPA14_ Up_f	ATCCGCACCGCCTGATCG	Forward primer annealing 380 bp upstream of the *hfq* gene for verification of deletion (13)
32	TS2hfqPA14_Down_r	GGGTGGCGAACAACTGGT	Reverse primer annealing 307 bp downstream of the *hfq* gene for verification of deletion (13)
33	qErsA_for	CTTCTTGAGCCCTTCGATGC	RT-qPCR primers for *ersA* (expected amplicon: 112 bp)
34	qErsA_rev	GAGCTTCGTATGGGGAGGG	
35	qAlgC_1062_RT_fw	CCAGGATCAGCGTGACAGC	RT-qPCR primers for *algC* (expected amplicon: 148 bp) (3)
36	qAlgC 1209 RT_rv	GATGTTGCCTTCGCCCCATT	
37	qVacJ 20 RT_fw	ACTGGATCGAACGTAGTGCC	RT-qPCR primers for *mlaA* (expected amplicon: 145 bp)
38	qVacJ 164 RT_rv	GCATAGGTGTCCAGGGTGTC	
39	qOprD_int_FW	AAGACCATGCTGAAGTGG	RT-qPCR primers for *oprD* (expected amplicon: 201 bp)
40	qOprD_int_RV	GTAGGTGGCATAGAGTTC	
41	16S_f	TGTCGTCAGCTCGTGTCGTGA	RT-qPCR primers for 16S (expected amplicon: 130 bp)
42	16S_r	ATCCCCACCTTCCTCCGGT	

[a] Lowercase: sites for restriction enzymes. Bold: T7 promoter sequence. Underlined: Shine-Dalgarno sequence. Bold Underlined: stop codon

References

1. Stover CK, Pham XQ, Erwin AL, Mizoguchi SD, Warrener P, Hickey MJ, Brinkman FS, Hufnagle WO, Kowalik DJ, Lagrou M, Garber RL, Goltry L, Tolentino E, Westbrock-Wadman S, Yuan Y, Brody LL, Coulter SN, Folger KR, Kas A, Larbig K, Lim R, Smith K, Spencer D, Wong GK, Wu Z, Paulsen IT, Reizer J, Saier MH, Hancock RE, Lory S, Olson MV. 2000. Complete genome sequence of *Pseudomonas aeruginosa* PAO1, an opportunistic pathogen. Nature 406:959-64.

2. Sonnleitner E, Hagens S, Rosenau F, Wilhelm S, Habel A, Jager KE, Blasi U. 2003. Reduced virulence of a hfq mutant of *Pseudomonas aeruginosa* O1. Microb Pathog 35:217-28.

3. Ferrara S, Carloni S, Fulco R, Falcone M, Macchi R, Bertoni G. 2015. Post-transcriptional regulation of the virulence-associated enzyme AlgC by the σ22-dependent small RNA ErsA of *Pseudomonas aeruginosa*. Environmental Microbiology 17:199-214.

4. Pawar SV, Messina M, Rinaldo S, Cutruzzola F, Kaever V, Rampioni G, Leoni L. 2016. Novel genetic tools to tackle c-di-GMP-dependent signalling in *Pseudomonas aeruginosa*. J Appl Microbiol 120:205-17.

5. Sasaki I, Bertani G. 1965. Growth abnormalities in Hfr derivatives of *Escherichia coli* strain C. J Gen Microbiol 40:365-76.

6. de Lorenzo V, Timmis KN. 1994. Analysis and construction of stable phenotypes in gram-negative bacteria with Tn5- and Tn10-derived minitransposons. Methods Enzymol 235:386-405.

7. Finan TM, Kunkel B, De Vos GF, Signer ER. 1986. Second symbiotic megaplasmid in *Rhizobium meliloti* carrying exopolysaccharide and thiamine synthesis genes. J Bacteriol 167:66-72.

8. Qiu D, Damron FH, Mima T, Schweizer HP, Yu HD. 2008. PBAD-based shuttle vectors for functional analysis of toxic and highly regulated genes in *Pseudomonas* and *Burkholderia* spp. and other bacteria. Appl Environ Microbiol 74:7422-6.

9. Delvillani F, Sciandrone B, Peano C, Petiti L, Berens C, Georgi C, Ferrara S, Bertoni G, Pasini ME, Dehò G, Briani F. 2014. Tet-trap, a genetic approach to the identification of bacterial RNA thermometers: Application to *Pseudomonas aeruginosa*. RNA 20:1963-1976.

10. Rybtke, M.T., Borlee, B.R., Murakami, K., Irie, Y., Hentzer, M., Nielsen, T.E., et al. (2012) Fluorescence-based reporter for gauging cyclic di-GMP levels in *Pseudomonas aeruginosa*. Appl Environ Microbiol 78: 5060–5069.

11. Corcoran CP, Podkaminski D, Papenfort K, Urban JH, Hinton JC, Vogel J. 2012. Superfolder GFP reporters validate diverse new mRNA targets of the classic porin regulator, MicF RNA. Mol Microbiol 84:428-45.

12. Kovach ME, Elzer PH, Hill DS, Robertson GT, Farris MA, Roop RM, 2nd, Peterson KM. 1995. Four new derivatives of the broad-host-range cloning vector pBBR1MCS, carrying different antibiotic-resistance cassettes. Gene 166:175-6.

13. Carloni S, Macchi R, Sattin S, Ferrara S, Bertoni G. 2017. The small RNA ReaL: a novel regulatory element embedded in the *Pseudomonas aeruginosa* quorum sensing networks. Environmental Microbiology 19:4220-4237.

14. Martinez-Garcia E, de LV. 2011. Engineering multiple genomic deletions in Gram-negative bacteria: analysis of the multi-resistant antibiotic profile of *Pseudomonas putida* KT2440. Environmental microbiology 13:2702-16.

15. Ferrara S, Brugnoli M, de Bonis A, Righetti F, Delvillani F, Dehò G, Horner D, Briani F, Bertoni G. 2012. Comparative profiling of *Pseudomonas aeruginosa* strains reveals differential expression of novel unique and conserved small RNAs. PLoS ONE 7.
